# Recycling and Reprocessing of Flax/Acrylic Biocomposites: A Dissolution Method Using Acetone as Solvent and Water as Non-Solvent

**DOI:** 10.3390/polym18202466

**Published:** 2026-10-09

**Authors:** Maurice Sager Sawalha, Aditya Prakash Shembekar, Arun Kumar Alapati, Aslıhan Kabaş, Ali Khodayari, David Seveno, Eleonora Ricci, Dipa Ray

**Affiliations:** 1 School of Engineering, Institute for Materials and Processes, The University of Edinburgh, Sanderson Building, Robert Stevenson Road, Edinburgh EH9 3FB, UKericci@ed.ac.uk (E.R.); 2 Laboratory of Complex Surfaces and Interfaces, Department of Materials Engineering, KU Leuven, Kasteelpark Arenberg 44, 3001 Leuven, Belgiumali.khodayari@kuleuven.be (A.K.); david.seveno@kuleuven.be (D.S.)

**Keywords:** flax/acrylic composites, composites recycling, dissolution, water non-solvent, thermoplastic biocomposites, second-generation composites

## Abstract

This study explores the second-generation biocomposites utilising flax fabric reinforcement with liquid thermoplastic Elium^®^ (acrylic) resin. The surface tension and wettability of the liquid acrylic resin with flax fabric were characterised through experimental techniques. Subsequent manufacturing of second-generation flax fibre/Elium^®^ laminates involved distinct reprocessing routes incorporating varying levels of reclaimed materials, with the natural fibre pre-form preserved and reused. A dissolution process using acetone as solvent and water as non-solvent enabled recovery of the acrylic polymer matrix. The residual polymer remaining on the flax fibres after dissolution served as a sizing agent in the second-generation laminates, resulting in improved interfacial bonding. After normalisation to a common fibre volume fraction, the second-generation infused laminate demonstrated a 6.5% increase in flexural strength, a 16% increase in flexural modulus, and an 11.4% increase in short beam strength compared to its virgin counterpart. Molecular simulations further elucidated the interactions between flax fibre, acrylic monomer, and acrylic polymer. These findings demonstrate a viable pathway for the production of circular acrylic matrix based thermoplastic biocomposites with significant industrial potential.

## 1. Introduction

The field of advanced composites is under pressure to develop more sustainable alternatives to high-performance materials in order to align with net zero initiatives. The European Union (EU) and the United Kingdom have both committed to achieving climate neutrality by 2050 through the European Climate Law and the UK Climate Change Act, with the EU also targeting at least a 55% reduction in net greenhouse gas emissions from 1990 levels by 2030 [1,2]. These targets are ambitious, but the industry is changing quickly in response. The use of natural fibres in composites is becoming more popular in the automotive, marine, and aerospace industries, mainly as alternatives to glass fibre; however, end-of-life research remains needed in order to further reduce the carbon footprint associated with manufacturing [3].

Combining natural fibres with liquid thermoplastic resins such as Elium^®^ poses a sustainable solution. Composites made with liquid thermoplastic resins have the advantage of being manufactured through the same routes as traditional thermoset resins, such as vacuum assisted resin transfer moulding (VARTM), but can also be reprocessed as thermoplastics [4]. In addition, the polymer matrix can be separated by chemical dissolution, allowing for recovery of the fibres and acrylic polymer at room temperature [5].

Although natural fibres are biodegradable and renewable [6], their biodegradability is negated when combined with synthetic materials. Even after separation, the fibres contain residuals that prevent biodegradability, so reuse of the composite constituents while preserving fibre integrity is important. While research in the field of recyclable biocomposites exists [7,8], many experiments tend to isolate the testing of recyclable matrices and their fibre reinforcements rather than covering the complete lifecycle process. While the recycling of acrylic and synthetic fibres is better documented [9,10], there remains a gap in the full recycling of acrylic composites based on natural fibres.

Allagui et al. investigated the recycling of woven flax fibre/acrylic (Elium^®^) composites by reprocessing chopped waste material via compression moulding [11]. The resulting laminates exhibited a substantial reduction in tensile strength and failure strain compared with the virgin material, with further degradation observed over successive recycling cycles due to progressive fibre shortening. Fibre length plays a critical role in composite performance, with continuous fibres providing superior tensile and flexural properties [12]. These findings highlight the importance of recycling approaches that preserve fibre length and pre-form shape, although maintaining fibre orientation during certain processes such as compression moulding remains challenging [13]. In contrast, Obande et al. [14] reprocessed intact liquid-infused glass fibre/Elium^®^ laminates by hot-press thermal reshaping, finding a 13% increase in flexural strength and a 9% increase in modulus relative to the as-infused material. This demonstrates that a secondary thermal consolidation step can improve the properties of a laminate provided that the reinforcement’s integrity is preserved.

Frej et al. [9] demonstrated closed-loop recycling of acrylic-based carbon fibre composites by recovering the Elium^®^ acrylic matrix via thermal depolymerisation (pyrolysis) under an inert nitrogen atmosphere, followed by purification and re-polymerisation. This approach required elevated temperatures (350–450 °C) to depolymerise the acrylic resin, enabling preservation of the carbon fibre pre-form for remanufacture. While effective for carbon fibre systems, such high-temperature and inert-atmosphere processes are unsuitable for natural fibres, which are prone to thermal degradation at significantly lower temperatures (180–230 °C) [15,16]. This highlights the need for alternative lower-temperature recycling routes for natural fibre thermoplastic composites. A study by C. Tschentscher et al. compared the dissolution process of carbon fibre (CF)/Elium^®^ composites using multiple solvents, finding that acetone was the most effective solvent for dissolving an Elium^®^ 150 matrix in the shortest amount of time compared to xylene, acetophenone, and ethyl acetate [17]. The dissolved matrix was recovered either by evaporating the solvent or using another synthetic nonsolvent to precipitate the acrylic polymer. However, to our knowledge, dissolution has not been sufficiently studied with flax fibre/acrylic composites, and water has not previously been used as a non-solvent to recover the dissolved acrylic matrix during the dissolution process with Elium^®^ composites.

The successful reuse of reclaimed fibres depends on how they interact with the resin, undergo wetting, and develop strong interfacial bonds. A major limitation of combining natural fibres with synthetic polymers is the inherent hydrophilicity of the fibre surface, which leads to poor compatibility with comparatively hydrophobic polymer matrices. As a result, flax and other plant fibres are often chemically treated, for example by alkalisation or silane sizing, to improve their impregnation and fibre–matrix adhesion; such treatments have been shown to improve adhesion, as indicated by increased interfacial strength up to 22% for flax fibre/Elium^®^ interfaces [4,18].

Molecular simulations can provide insight into the behaviour at the fibre/matrix interface, which can in turn help to elucidate macroscopic behaviour and phase compatibility. For example, Konrad et al. [19] performed molecular simulations to characterise the interaction forces at the interface between crystalline cellulose and epoxy resins, which were found to be significantly weaker compared to the inner cellulose region. In particular, the hydrogen bond network was one order of magnitude less dense and the average length of hydrogen bonds was double at the interface [19]. Analogous behaviour was observed by Ren et al. for a polylactic acid (PLA)–cellulose interface [20], which they ascribed to poor macro-scale adhesion between the PLA and cellulose. Molecular simulation can provide interesting insights into how a hydrophilic flax fibre (cellulose) surface interacts with an acrylic monomeric resin and an acrylic polymer as well as how this interaction changes when the flax fibre surface retains a residual coating of acrylic polymer after being recovered from a dissolution process; we study these interactions in the present work.

A study by W. Obande et al. [21] evaluated the properties of thick thermoplastic laminates made with multi-stage liquid resin infusion, finding an increase in short beam strength (SBS) compared to a single stage infusion. Their results suggest that the increase in SBS can be attributed to the formation of semi-interpenetrating polymer networks (semi-IPNs). Such semi-IPNs develop when new liquid resin dissolves the surface of a pre-consolidated acrylic. This promotes diffusion of the infused monomers lying between the existing polymer chains, forming a semi-IPN. The resulting structure forms a strong interface, as polymer chains become entangled across the boundary between the previous and newly consolidated layers [21]. The manufacturing process of the second-generation laminate rFF/vElium^®^ is similar, involving fabrics coated in a layer of residual acrylic polymer. The formation of semi-IPNs between methyl methacrylate (MMA) and poly(methyl methacrylate) (PMMA) prompts further research into the interaction between these two phases.

The novelty of this study is in the use of water as a non-solvent and in the preservation of the woven flax fibre pre-form during the proposed recycling methods. This approach has the potential to significantly reduce emissions associated with the synthesis and disposal of alternative chemical non-solvents such as methanol or ethanol [22]. Although water has previously been used to precipitate and agglomerate polypropylene (PP) and poly(ethylene terephthalate) (PET) solutions in chemistry applications, its use in composite recycling has not been reported [23]. This presents a significant opportunity for using water in an industrially scalable recycling process for acrylic-based thermoplastic composites.

This study accordingly investigates the implications of flax/acrylic composite recycling and its impact on reclaimed materials, manufacturing, and the properties of the resulting second-generation composites. Molecular simulations were conducted to qualitatively compare the relative interfacial affinity of MMA monomer (liquid Elium^®^), PMMA (polymerised Elium^®^), and the flax fibre fabric (modelled with a representative cellulose phase), while the wetting dynamics of the flax fabric and Elium^®^ were measured using the Wilhelmy and pendant drop methods. The test results show possible full circularity of flax/Elium^®^ composites and high potential for industrial adoption.

## 2. Materials and Methods

### 2.1. Materials and Manufacturing

The reinforcing fabric used in this study was 360 g/m^2^ 2/2 twill woven flax fabric (Eco-Technilin, Valliquerville, France) with a density of 1.45 g/cm^3^. All laminates were manufactured with four plies of fabric layered in a cross-ply configuration [0/90/0/90] to adjust for changes in weft and warp strength (51 wt% warp 49 wt% weft; see Table A1). Elium^®^ 191 XO/SA was used as the acrylic matrix in laminate manufacture and Elium^®^ 151 XO/SA was used in wettability studies, both sourced from Arkema (La Défense, France). Both are low-viscosity (cP @ 25 °C = 100 mPa s) and low-exotherm thermoplastic resins that are infusible at room temperature. XO and SA parts were mixed at 50 weight% (wt%) per part, and methyl ethyl ketone peroxide (MEKP) Butanox-M50 (Nouryon, Amsterdam, The Netherlands) at 3 wt% was used to initiate the free radical polymerisation of the resin. No post-curing process was needed. Marbocote 227 CE (Marbocote Ltd., Northwich, UK) was used as a release agent for the vacuum-assisted resin transfer moulding (VARTM) method and Loctite 770-NC Frekote (Henkel, Düsseldorf, Germany) for the compression moulding process. Dissolution was performed with 99.5% purity technical acetone (Fisher Chemical 1090; Fisher Scientific, Loughborough, UK), while water was used to precipitate and reclaim the polymer matrix.

In this study, four flax fibre (FF)/acrylic (Elium^®^) composites were manufactured, as shown in Table 1.

### 2.2. First-Generation Laminate Manufacture

#### 2.2.1. Manufacturing v(FF/Elium^®^)

Two identical first-generation laminates were prepared with standard VARTM. Prior to infusion, FF plies were dried at 105 °C for 4 h to partially remove moisture [16]. The infused laminates were left to polymerise for 24 h at room temperature before demoulding.

#### 2.2.2. Manufacturing v(FF/Elium^®^)CM

The v(FF/Elium^®^)CM was postprocessed from infused virgin laminate v(FF/Elium^®^). The laminate sample was compression-moulded in a heated platen press (Lab450P, Pinette Emidecau Industries, Chalon-sur-Saône, France). It was heated to 150 °C for 5 min, then ≈20 bar was applied for 10 min.

### 2.3. Second-Generation Laminate Manufacture

#### 2.3.1. Recycling—Separating the Constituents

**Figure 1:**
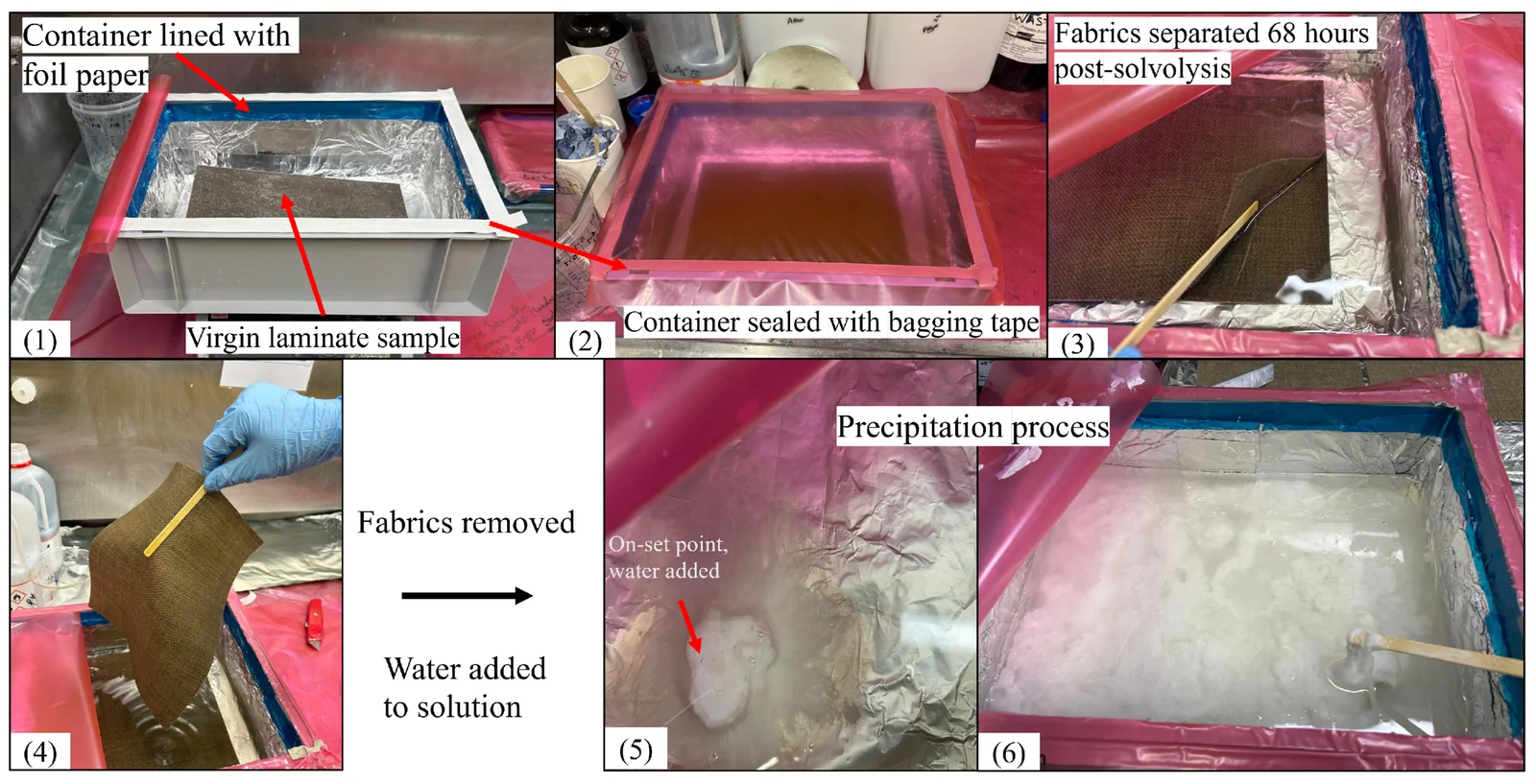
Dissolution and polymer recovery process: (**1**) virgin laminate placed in a foil-lined container, (**2**) acetone added and container sealed with bagging tape, (**3**) fabrics visibly separating after 68 h, (**4**) reclaimed fabrics removed from solution, (**5**) onset of polymer precipitation following the addition of water, and (**6**) polymer precipitation process nearing completion.

Chemical dissolution was used to separate the reinforcing FF fabric and acrylic polymer. In this study, dissolution was performed using acetone, which is the most effective solvent, as reported in ref. [17]. The process was carried out with two different polymer/solvent (P/S) ratios, one at 1:8 and another at 1:14. In both cases, the fabrics were removed from the resultant solution after 68 h and set to dry in open air. Polymer residue was visible on the fibre surfaces with the naked eye. The process can be seen in Figure 1. Details on the dissolution process and material yield can be found in Table A2. The mass of reclaimed fabrics was 23.3% (1:8 P/S) and 10.1% (1:14 P/S) higher than the mass of virgin fabrics, respectively, due to residual polymer remaining on the fibre surface after dissolution.

#### 2.3.2. Polymer Precipitation

**Figure 2:**
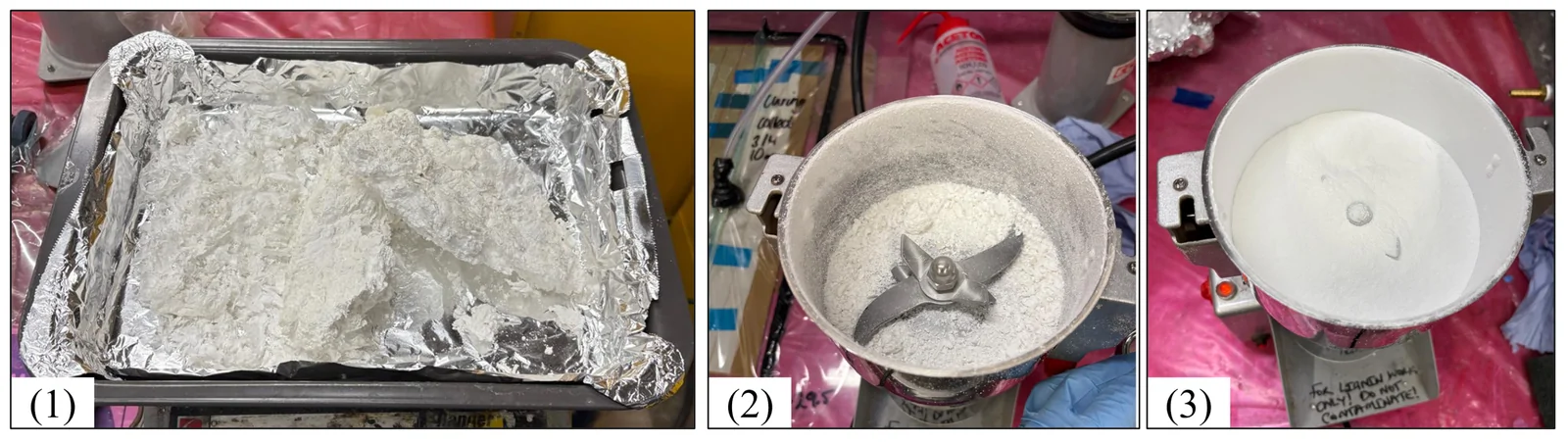
Reclaimed polymer processing: (**1**) dried coalesced polymer post-precipitation, (**2**) ground polymer flakes, and (**3**) finely ground reclaimed polymer powder.

Precipitation was performed on the solution with a 1:14 P/S ratio. The polymer was dried and then ground into a fine powder using a coffee grinder. Precipitation is shown in steps 5–6 in Figure 1, while polymer grinding and drying are shown in Figure 2. Although not done in this study, acetone can be separated from the aqueous solution using distillation, as presented in various studies [24,25,26,27]; however, this requires further exploration.

#### 2.3.3. Manufacturing rFF/vElium^®^

The reclaimed FF was re-infused with virgin liquid resin (Elium^®^ 191) using standard VARTM, as was done for the first-generation (virgin) laminates. The reclaimed plies of FF were then laid in the same order as for the virgin laminate.

#### 2.3.4. Manufacturing r(FF/Elium^®^)CM

**Figure 3:**
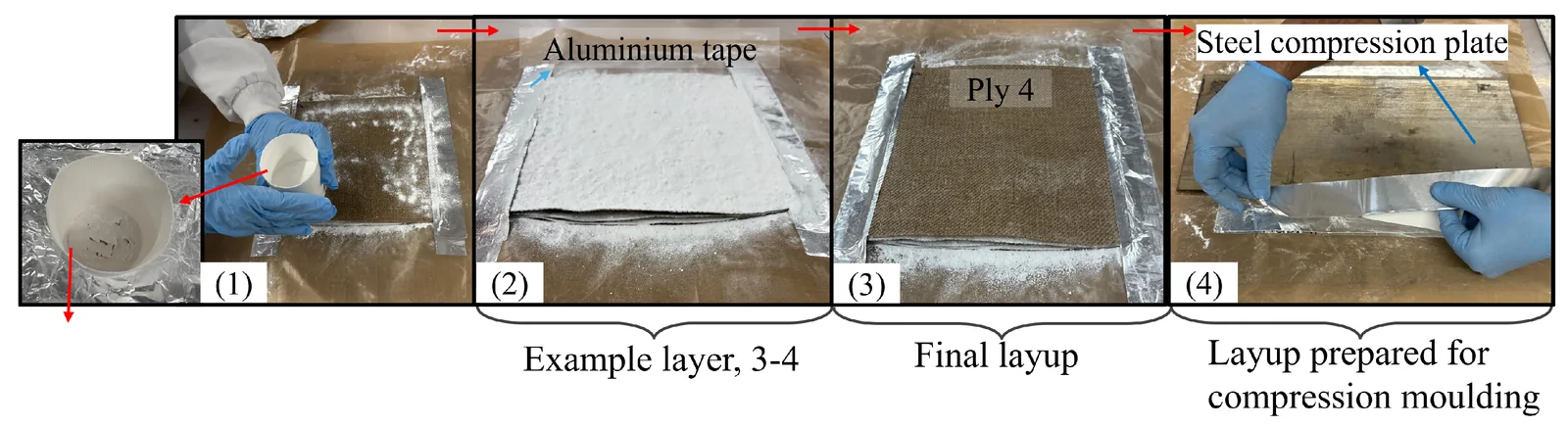
Preparation of r(FF/Elium^®^)CM laminate using reclaimed polymer powder: (**1**) polymer powder deposited using a paper cup, (**2**) intermediate layers (plies **3**,**4**) showing polymer distribution, (**3**) final layup with fourth ply applied, and (**4**) laminate assembly positioned between steel compression plates for compression moulding.

r(FF/Elium^®^)CM was manufactured by compression moulding of reclaimed FF and reclaimed acrylic polymer powder. The target fibre volume fraction of the laminate was 40%. Manufacturing involved stacking the four plies of reclaimed fabric and depositing powder between each layer (see Figure 3). A 2 mm thick frame was used and compression moulding was performed on the same heated platen press. The layup was compressed at ≈35 bar and 170 °C for 15 min. The cycle time was kept short to avoid fibre degradation.

### 2.4. Fibre Volume Fractions

The fibre volume fraction was determined gravimetrically from the mass of dry fabric and the mass of the consolidated laminate, together with the constituent densities; the equations used are given in Appendix C. The calculation was performed on three samples of each laminate and averaged. The resulting fibre volume fractions are given in Table 2. As no void or pore correction was applied, the values in Table 2 represent nominal fibre volume fractions.

## 3. Testing

### 3.1. Surface Tension Measurement

Surface tension of the liquid Elium^®^ resin was measured using both the pendant drop method [28] (OCA 50) and the Wilhelmy method (DCAT 25), both supplied by DataPhysics Instruments GmbH (Filderstadt, Germany). In the pendant drop method, 5 μL of Elium^®^ was dispensed using a syringe with a needle of outer diameter 0.91 mm, inner diameter 0.61 mm, and length 38.1 mm. Surface tension was then calculated by analysing the droplet profile using SCA20 software (Version 6.2.26 build 6026, DataPhysics Instruments GmbH, Filderstadt, Germany).

In the Wilhelmy method, a freshly flame-activated platinum plate with high surface energy (width = 0.2 mm, length = 19.9 mm, thickness = 0.8 mm) was immersed 4 mm into the Elium^®^. Calibration was performed using water, so the surface tension is well-established. The procedure was repeated five times; after each trial, the plate was cleaned with acetone, rinsed with pure water, and dried with heat. Due to the relatively high viscosity of Elium^®^, the plate was immersed at a speed of 0.04 mm/s to minimise any effects associated with solidification, as Elium^®^ exhibits noticeable viscosity changes as soon as the MMA monomers begin to evaporate. Force data were recorded and analysed using DCATS software (Version 4.3.58.250, DataPhysics Instruments GmbH, Filderstadt, Germany).

Static and dynamic contact angle measurements were conducted using a 5 μL Elium^®^ droplet deposited onto a glass slide, using the same syringe dimensions described above. The glass slides were cleaned with ethanol and hexane, then dried in an oven for 1 h before each experiment. To obtain advancing–receding hysteresis of the droplet, the stage was tilted to 90° at a speed of 12.06°/s, with contact angles acquired at each tilting step (OCA 50, DataPhysics Instruments GmbH).

Infiltration of virgin flax fabrics was measured using the modified Washburn approach (DCAT 25, DataPhysics Instruments GmbH). Virgin flax fabrics (2 cm × 9 cm) were wrapped and positioned inside a capillary tube. Each sample was tested three times. After each experiment, the tube was cleaned with isopropanol in an ultrasonic bath for 15 min, followed by brushing and washing with soap.

All experiments were performed in an acclimatised room with controlled temperature (23 ± 0.5 °C) and humidity (40 ± 5%).

### 3.2. Testing of Composites

Flexural strength and modulus were tested according to ASTM D7264 on an Instron 3369 (Instron, Norwood, MA, USA) using a 10 kN load cell. Midspan deflection was assumed to be equal to the crosshead extension. Short beam strength tests followed ASTM D2344 using the same Instron 3369 and 10 kN load cell as above. A span length/thickness ratio of 4:1 was used, and the crosshead speed was set to 1 mm/min for all samples. To allow for direct comparison between results, the measured flexural strength, flexural modulus, and short beam strength values were normalised to the fibre volume fraction of the virgin laminate v(FF/Elium^®^) (27.37%).

Dynamic mechanical analysis (DMA) was used to measure the glass transition temperatures of the first- and second-generation laminate specimens in accordance with ASTM 4065. The test was performed on a TA Instruments Discovery DMA 850 (TA Instruments, New Castle, DE, USA) with an amplitude of 20 µm and frequency of 1 Hz on a 3-point bend setup. The temperature ramp was set to 5 °C/min between ambient and 160 °C.

Scanning electron microscopy (SEM) was used to analyse fracture surfaces and the surfaces of both virgin and reclaimed fibres. All SEM samples were sputter coated with 35 nm Au and analysed using a Hitachi TM4000Plus (Hitachi High-Tech Corporation, Tokyo, Japan) at an accelerating voltage of 15 kV. Fracture samples were cut using a dry saw, and did not require any heat treatment post-cutting.

### 3.3. Molecular Modelling

To probe the interfacial behaviour of the composite at a molecular level, an atomistic model consisting of three layers was built: one of amorphous cellulose, one of PMMA to represent the residual acrylic coating, and one of liquid methyl methacrylate (MMA) to represent the liquid monomeric resin. The three interfaces present in such a system can be considered ideal representations of different processing steps in the recycled composite material life cycle: cellulose/MMA represents the step of infusing the virgin fabric with the liquid acrylic monomeric resin, PMMA/MMA represents the step of reinfusing the reclaimed fabric (rFF) with the liquid acrylic monomeric resin, and cellulose/PMMA represents the fibre–coating interactions of rFF itself.

Cellulose is considered a simplified representation of the flax fibre, being its main constituent, and the amorphous phase was chosen based on the assumption that it would have the most interaction with the resin at the interface. Due to the computational constraints associated with atomistic modelling, it was not possible to capture the full complexity of the flax fibre; this simplified representation was chosen for preliminary validation of the force field and equilibration protocol as well as for qualitative analysis of interactions with the monomer liquid and acrylic coating. The presence of crystallinity in the cellulose phase would have a major influence on the calculation of mechanical properties, but not on the intermolecular energies computed here. In future work, it would be interesting to explore the effect of a more realistic microstructure, but this is beyond the scope of the present work.

Generation of the initial configurations and force field assignment were performed using the MAPS platform (Version 5.0.0, Scienomics SARL, Paris, France) [29]. All subsequent energy minimisation and molecular dynamics runs were performed using LAMMPS (Version 2 Aug 2023, Sandia National Laboratories, Albuquerque, NM, USA) [30] on the Cirrus high-performance computing (HPC) cluster. All simulations were carried out in full atomistic detail at atmospheric pressure and 298 K. Postprocessing of the trajectories was performed using in-house Python scripts (Version 3.11, Python Software Foundation, Wilmington, DE, USA). Visualisations were created using VMD (Version 1.9.4, University of Illinois at Urbana-Champaign, Urbana, IL, USA) [31].

Different Class 1 and Class 2 force fields were preliminarily tested; ultimately, the sciPCFF force field, a variation of the polymer consistent force field (pcff) supplied along with the MAPS platform, was chosen, together with partial charges obtained using the Gasteiger calculation method [32]. This combination yielded the most accurate results for the density of the three systems considered when simulated separately. The simulations were carried out in full atomistic detail at atmospheric pressure and 298 K, with a cutoff of 12 Å for both electrostatic and van der Waals interactions. Long-range electrostatics were computed using a particle–particle particle–mesh (PPPM) solver [33] with a relative accuracy threshold of $10^{-4}$. A timestep of 1 fs was used in the preliminary single-phase simulations, while 0.5 fs was used in all stages for the three-phase system.

The three-phase system was constructed stepwise. First, the amorphous cellulose layer was built by packing 15 chains of 80 repeating units each into a 5 × 5 × 11 nm box (∼25,000 atoms, initial density 1.170 g/cm^3^) using the Amorphous Builder tool of MAPS. The system was confined in the *z* direction, while periodic boundaries were applied in the *x* and *y* directions. After performing energy minimisation for 5000 steps using the steepest descent method, flat walls were applied on the two faces perpendicular to the *z* axis (fix wall/lj93 command, with values of σ, ϵ, and cutoff of 1.0 Å, 1.0 kcal/mol, and 2.5 Å, respectively) and the system was shrunk in the *z* direction until it reached 80% of its initial length over the course of a 2 ns canonical (NVT) molecular dynamics run. The final density reached was 1.371 g/cm^3^, and the final box size was 5 × 5 × 9.4 nm. The system was further equilibrated for 3 ns in the NVT ensemble, with the walls still in place to preserve the interface.

After this run, the upper *z* face was moved to increase the box size to 5 × 5 × 19.5 nm, and 22 atactic PMMA chains of 80 repeating units were packed at a density of 1.18 g/cm^3^ into the created empty space using the Amorphous Builder tool of MAPS while keeping the system confined in the *z* direction. After performing energy minimisation for 5000 steps using the steepest descent method, flat walls were once again applied on the two faces perpendicular to the *z* axis and the system was equilibrated for 3 ns in the NVT ensemble.

The addition of the liquid MMA phase followed a similar procedure: the upper *z* face was moved to increase the box size to 5 × 5 × 30 nm, and 1450 molecules of methyl methacrylate were packed at a density of 0.945 g/cm^3^ into the created empty space using the Amorphous Builder tool of MAPS. From this point onward, periodic boundary conditions were applied in all three dimensions. After performing energy minimisation for 5000 steps using the steepest descent method, the system was equilibrated for 10 ns in the NVT ensemble, then for 10 ns in the isothermal-isobaric (NPT) ensemble, before a final a 10 ns production run in the NVT ensemble was performed.

## 4. Results and Discussion

### 4.1. Wettability Results

#### 4.1.1. Surface Tension of Acrylic (Elium^®^) Resin

**Figure 4:**
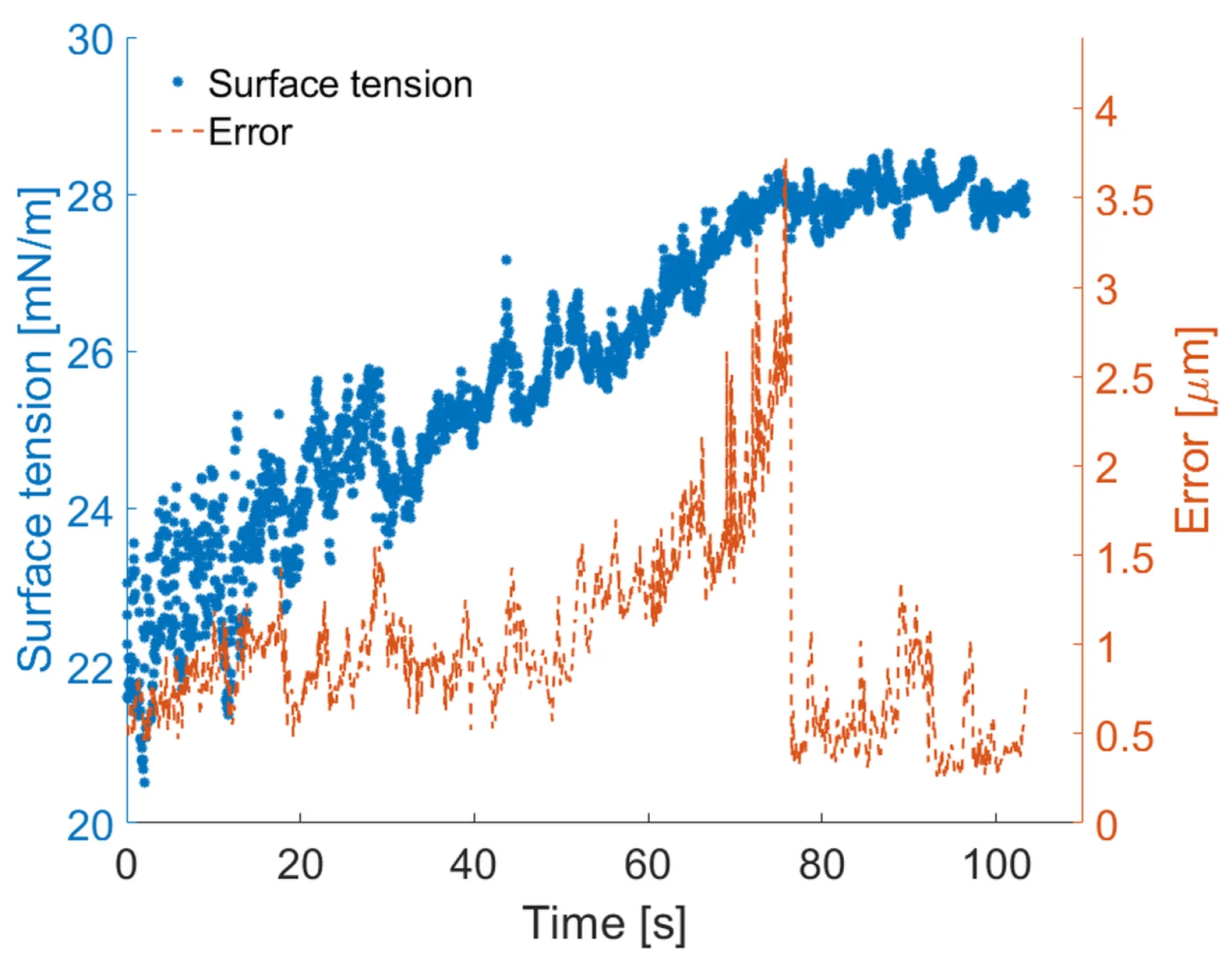
Estimation of the surface tension of Elium^®^ with the pendant drop method, and associated error. The surface tension reaches a plateau, accompanied by a decrease in the error.

The surface tension was first measured using the pendant drop method. During the measurement, the droplet volume gradually increased with time, and the surface tension was estimated at each step. At early times (up to 30 s), the droplet volume (and therefore mass) was too small for gravity to significantly influence its shape, meaning that the calculated surface tension values were not reliable. Once the droplet became sufficiently large for gravitational effects to compete with the Laplace pressure and the fitting error (defined as the mean distance between the droplet profile and the Laplace fit) remained low, the calculated surface tension stabilised (see Figure 4). Based on this stabilised region, the surface tension of Elium^®^ was determined to be 28.0 ± 0.2 mN/m.

**Figure 5:**
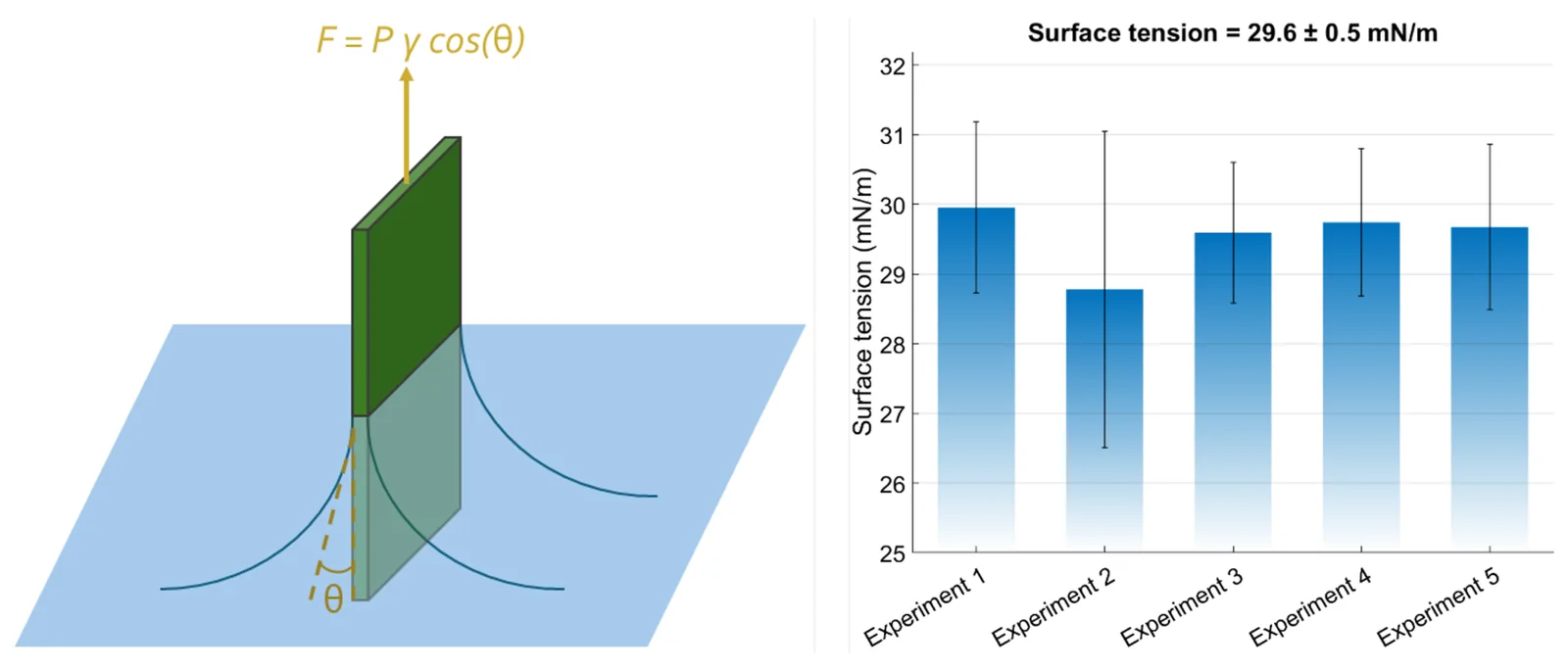
Surface tension estimations using the Wilhelmy method and tensiometer with a platinum plate. The measured values are slightly higher than the pendant drop calculations.

To validate this value, a second method, the Wilhelmy plate technique, was employed (Figure 5). Assuming that the receding contact angle approaches zero at the liquid–solid interface, such that cosθ=1, the capillary force simplifies to F=Pγ. The measured force was corrected for buoyancy effects using the known plate dimensions and the density of Elium^®^, then the resulting net capillary force was used to compute the surface tension. This method gave an average surface tension of 29.6 ± 0.5 mN/m.

Overall, the two techniques gave consistent results within experimental uncertainty. The measured values fall within the typical range reported for thermosetting resins, implying that the wetting and impregnation behaviour of Elium^®^ is comparable to that of many epoxy systems, and in some cases even slightly better [34,35].

#### 4.1.2. Wetting Dynamics of Acrylic (Elium^®^) Resin

**Figure 6:**
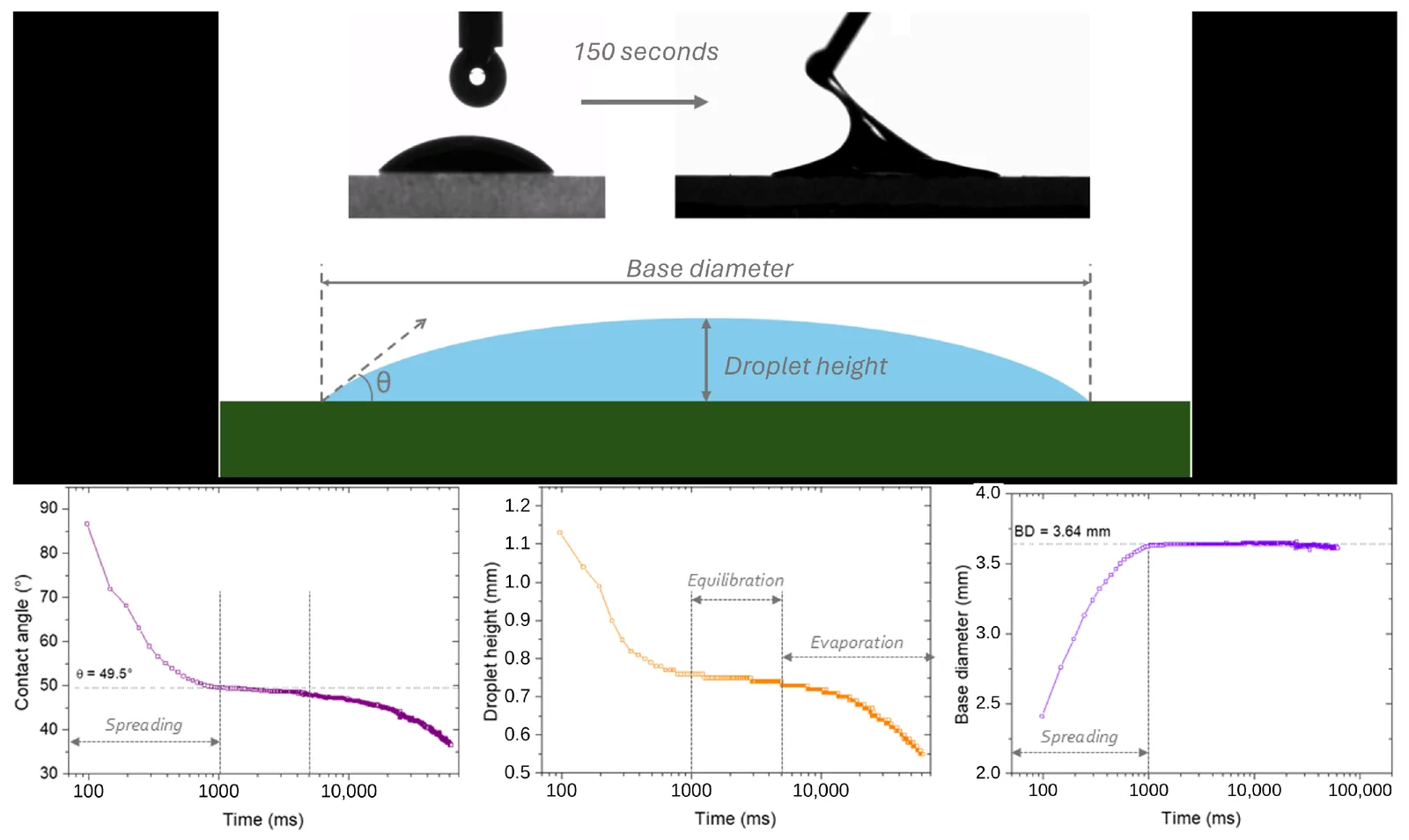
(**Top**) densification of Elium^®^ due to the evaporation of MMA monomers, (**Middle**) droplet parameters on substrate used for contact angle measurements, and (**Bottom**) evolution of contact angle, droplet height, and base diameter upon deposition on the glass slide.

The spreading dynamics of Elium^®^ were studied by analysing the profile of 5 μL droplets deposited on glass slides. Because the monomeric components of Elium^®^, primarily methyl methacrylate (MMA), evaporate rapidly upon exposure to ambient air, the liquid transitions to a more viscous state within a few seconds (Figure 6).

Immediately after deposition, the evolution of the droplet shape was monitored. The contact angle decreased rapidly and reached a steady value of approximately 49.5° within 1 s, indicating a hydrophilic wetting regime. The droplet then remained in a near-equilibrium spreading state for roughly 4 s, after which evaporation became dominant. The onset of evaporation is evident from the decrease in droplet height while the base diameter remains pinned, consistent with the classical constant-contact-radius evaporation mode [36]. This behaviour is attributed to rapid loss of the MMA-rich superficial liquid layer.

The evaporation behaviour can be qualitatively classified using the relative evaporation rate. Elium^®^’s MMA-containing phase corresponds to a value of 3.1 on the industrial evaporation rate scale reported by the Institut National de Recherche et de Sécurité (INRS), France [37], where *n*-butyl acetate is defined as the reference solvent with a rate of 1. According to this classification, evaporation rates below 0.8 are considered slow, rates between 0.8 and 3 are medium, and rates above 3 are fast [38]. Thus, Elium^®^ lies in the fast evaporation regime, consistent with the rapid droplet thinning observed experimentally.

**Figure 7:**
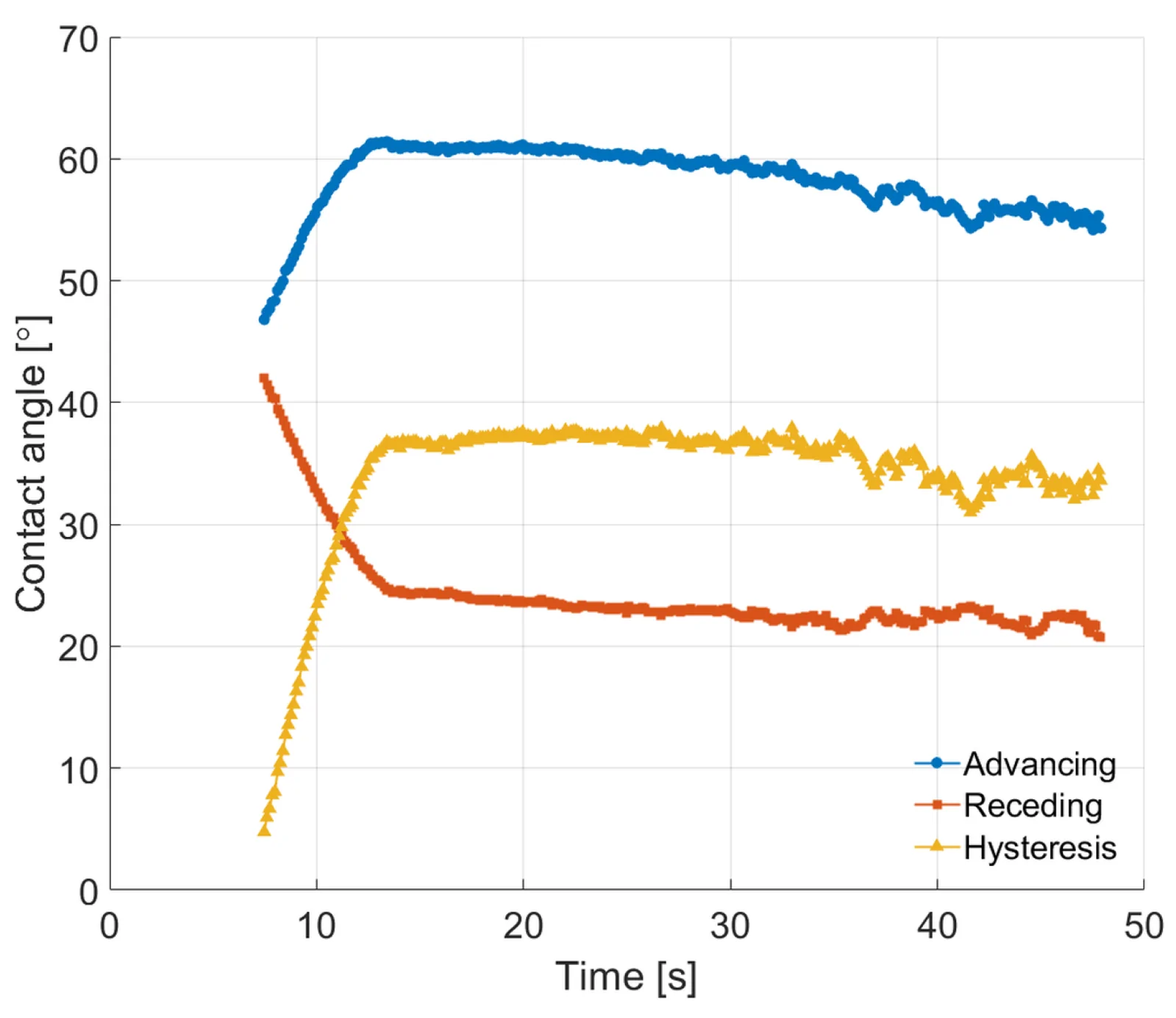
Obtained advancing and receding contact angle and the corresponding hysteresis. The hysteresis yields a value of 34° ± 6°, following a steady flow of the Elium^®^ droplet.

The measured difference between the advancing and receding contact angles (wetting hysteresis) was 34 ± 6°. Given the smoothness of the glass substrate, such a large hysteresis indicates strong contact-line pinning, suggesting that Elium^®^ interacts strongly with the substrate through either chemical heterogeneity or early-stage polymer–substrate interactions (Figure 7).

Combining the relatively high hysteresis observed here with the moderate viscosity of Elium^®^ (∼100 mPa s) and its low surface tension (∼29 mN/m), the resin is expected to wet the substrate effectively while also exhibiting strong adhesion. These properties suggest that Elium^®^ can achieve good surface coverage and contribute positively to impregnation quality. Nevertheless, it is important to note that although high hysteresis enhances resin retention and promotes adhesion, it does not necessarily translate into rapid impregnation in composite materials. Impregnation kinetics are governed primarily by viscosity and the advancing contact angle at the infiltration front, whereas hysteresis mainly reflects contact-line pinning and interfacial adhesion rather than flow rate.

#### 4.1.3. Wetting of Flax Fabrics with Acrylic (Elium^®^) Resin

**Figure 8:**
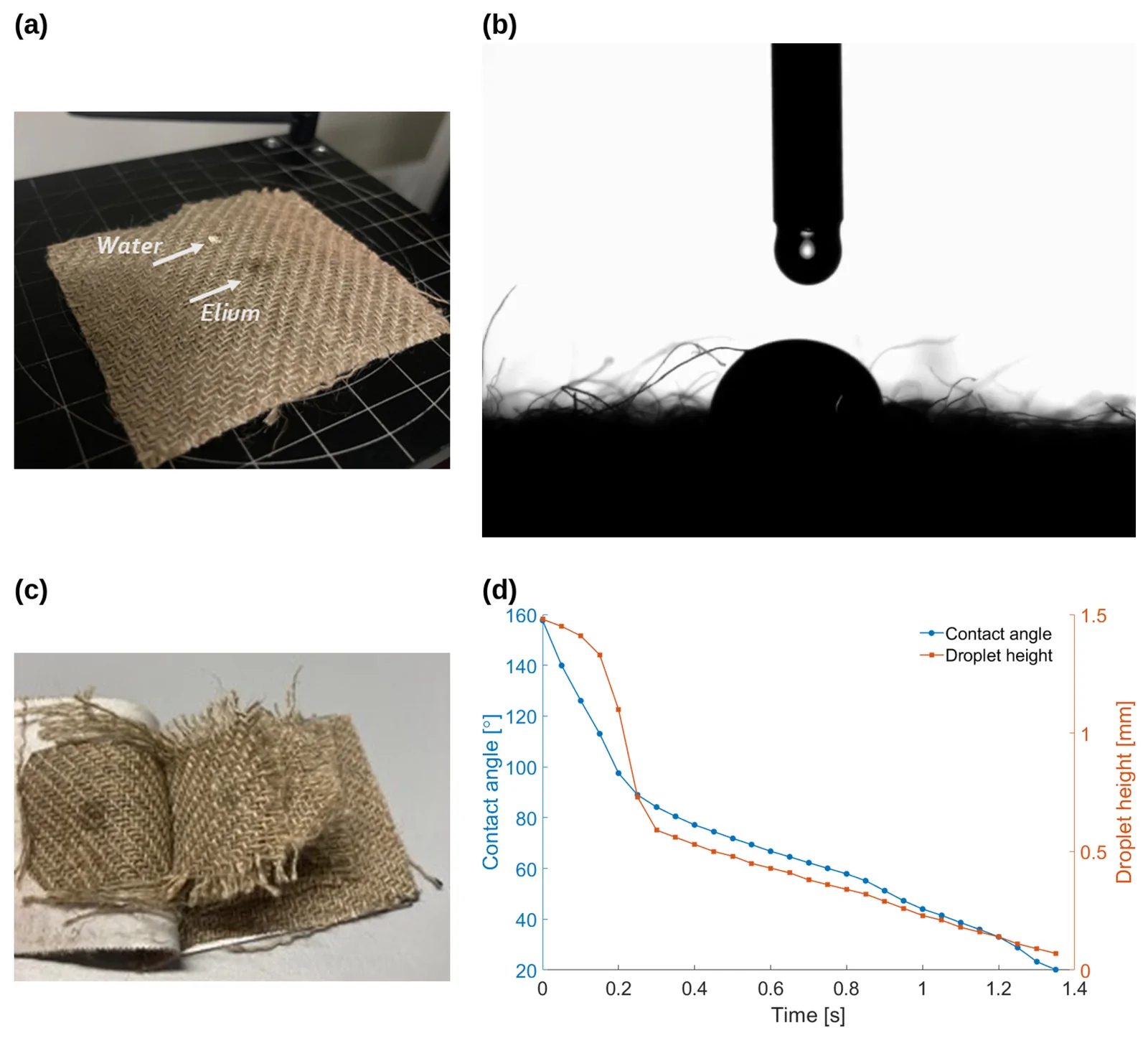
(**a**) Comparison of a water and an Elium^®^ droplet on the flax fabrics; (**b**) snapshot of a droplet deposited on the flax fibre before infiltration; (**c**) infiltration of Elium^®^ to the second layer of flax fabric stacks; and (**d**) the contact angle and Elium^®^ droplet height change in time during infiltration into a five-layer stack of flax fabric.

The wetting behaviour of flax fabrics shows remarkably fast penetration of Elium^®^. This was studied by depositing a 5 μL droplet onto a five-layer flax fabric stack affixed to an aluminium slab for mechanical stability and analysing the temporal evolution of the droplet height during infiltration. The fabrics exhibited strong absorbency towards Elium^®^, as illustrated by the comparison with water in Figure 8a. Notably, infiltration occurred predominantly in-plane within the first two layers, rather than progressing through the entire thickness of the stack. This is an interesting observation; in-plane infiltration was effectively instantaneous, whereas through-thickness infiltration would be expected to occur later during the vacuum-assisted infusion stage, when the layers are pressed into full contact.

Complete infiltration of the droplet occurred within approximately 1.4 s, as indicated by the droplet height approaching zero within this time. This rapid absorption highlights the affinity of Elium^®^ for flax fibres and the effectiveness of the fabric–resin interaction during the early stages of wetting.

**Figure 9:**
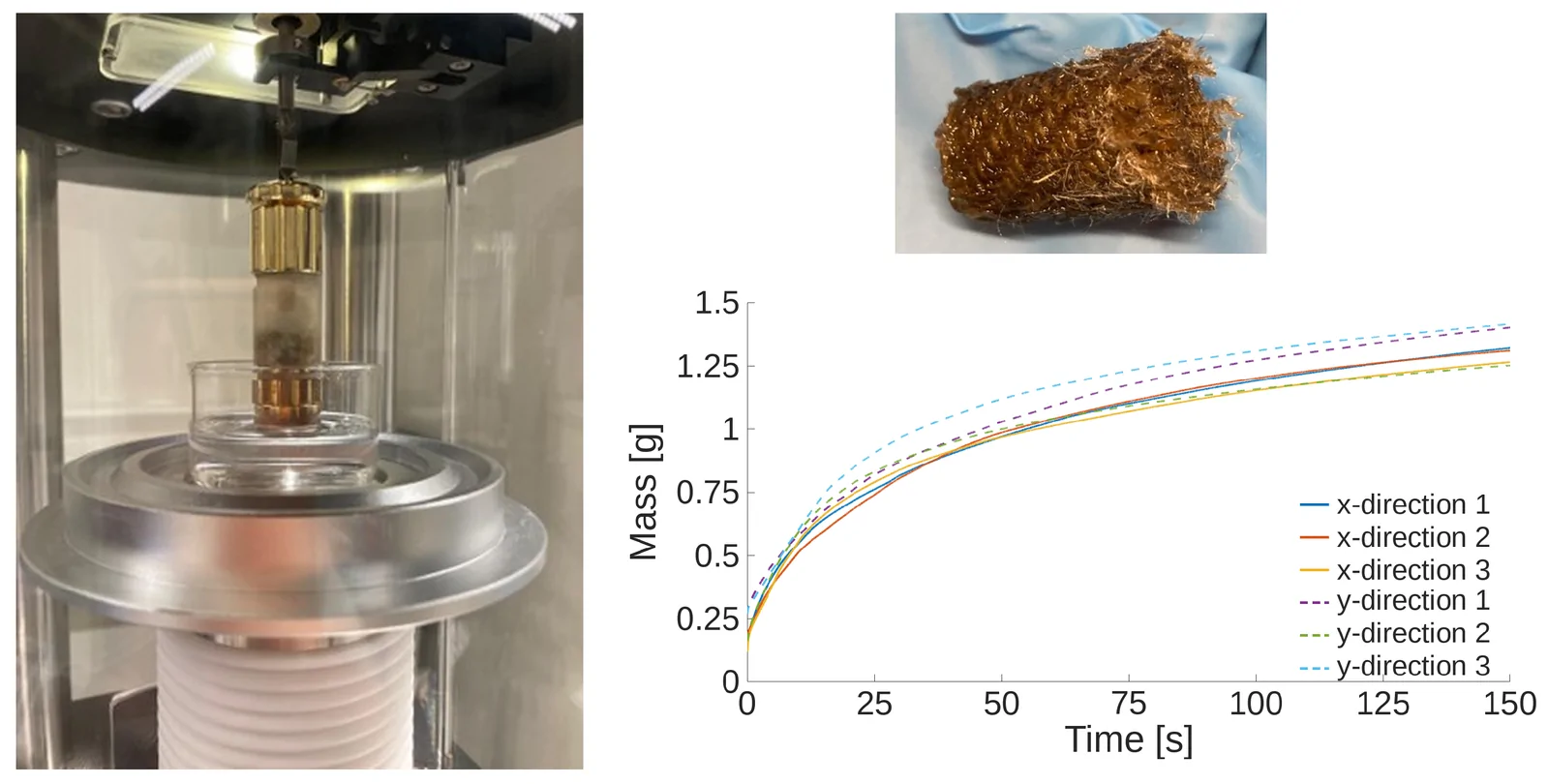
Impregnation and mass uptake measurements using DCAT on flax fabrics in two directions. Both directions show similar uptake properties.

**Figure A1:**
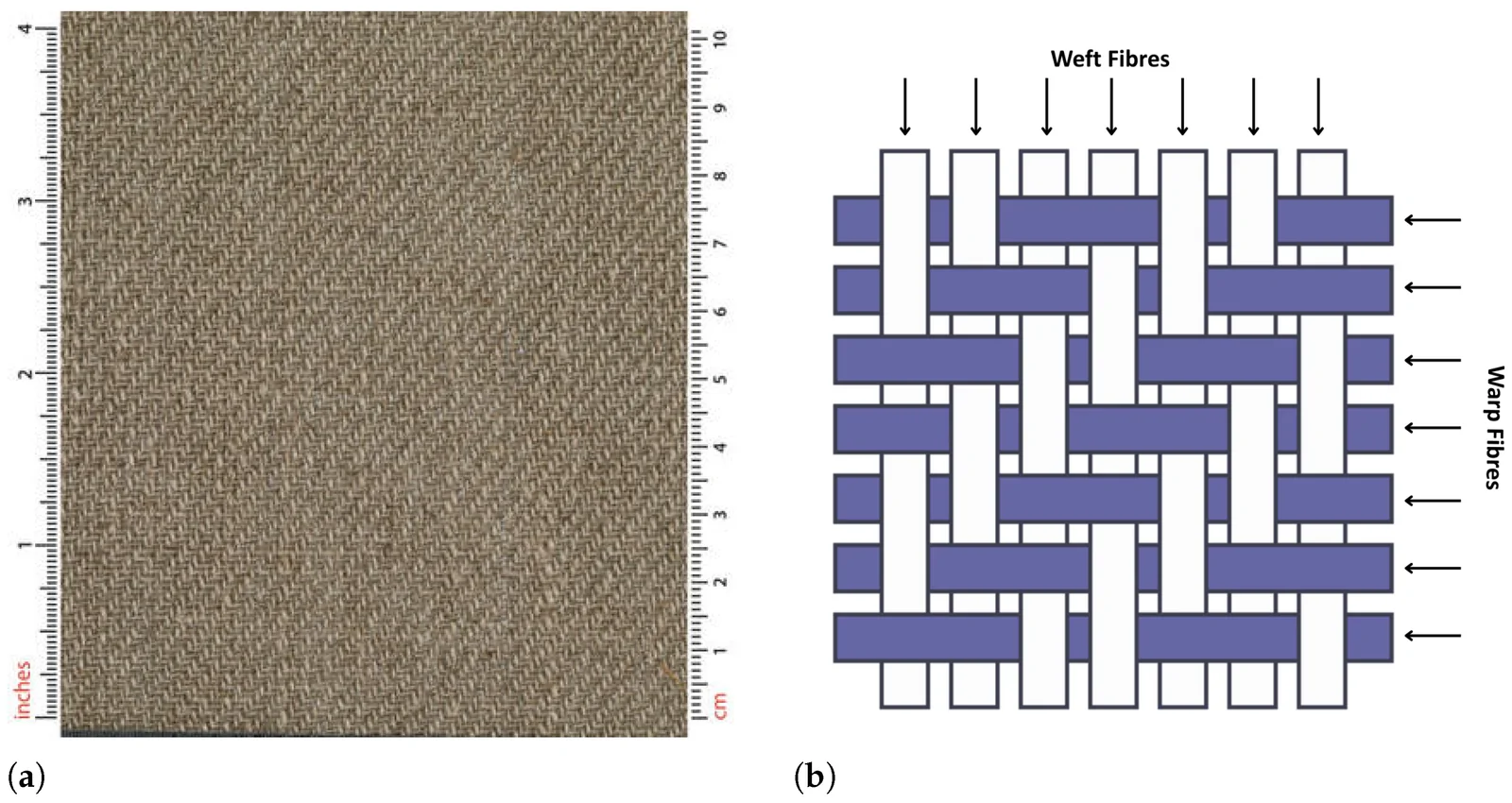
(**a**) Eco-Technilin 2/2 twill woven flax fabric (360 g/m^2^) and (**b**) weft and warp yarn orientation in a 2/2 twill weave [48].

To evaluate the mass uptake of the flax fabrics more quantitatively, 2 cm × 9 cm samples were cut in the x- and y-directions, corresponding to the warp and weft directions, respectively (as shown in Figure A1), then rolled and positioned inside a capillary tube, after which their impregnation behaviour was assessed using the DCAT 25. The results showed no significant difference between the two orientations over the first 150 s. The x-direction samples reached a mass uptake of 1.30 ± 0.03 g, while the y-direction samples reached 1.35 ± 0.09 g, indicating comparable levels of absorption in both directions (see Figure 9). This suggests that Elium^®^ impregnates the flax fabric effectively and uniformly regardless of the in-plane orientation.

#### 4.1.4. Implications of Wetting Behaviour for Fibre Impregnation

The above results, including the surface tension of Elium^®^, contact angle evolution, and capillary uptake by the fabrics, provide valuable insight into the impregnation behaviour during liquid composite moulding. The relatively low surface tension of Elium^®^ promotes spreading over the fibre surface and facilitates capillary penetration into the porous flax fabric structure, as observed in the Washburn experiments. Additionally, the rapid decrease in the contact angle upon droplet deposition indicates favourable initial wetting, an important parameter for the early stages of resin infusion (Figure 8d). However, the evaporation of monomers, i.e., the MMA, can increase the viscosity of the resin over time. Hence, it is important to maintain a relatively short exposure time before infusion in order to preserve resin mobility and ensure effective impregnation. The experiments also revealed pronounced contact angle hysteresis, indicating contact-line pinning and strong interactions between the resin and substrate. This could potentially contribute to resin retention and interfacial contact after impregnation. Nonetheless, hysteresis should not be interpreted as a direct indication of impregnation rate, since flow through the fabric is also controlled by other factors such as viscosity, fabric permeability, fibre packing, and applied pressure. The droplet’s rapid absorption into the flax fabric together with similar mass uptake in both the warp and weft directions, as depicted in Figure 8 and Figure 9, suggest that Elium^®^ can effectively impregnate the flax fabrics studied in this work. However, it must be noted that parameters such as laminate compaction and ply-to-ply contact remain crucial for achieving proper through-thickness impregnation during VARTM. Overall, the wetting results confirm that Elium^®^ is a suitable matrix for flax fabric impregnation, while also highlighting the importance of controlling resin exposure and processing conditions during infusion.

### 4.2. Composite Test Results

#### 4.2.1. Flexural Test Results

**Figure 10:**
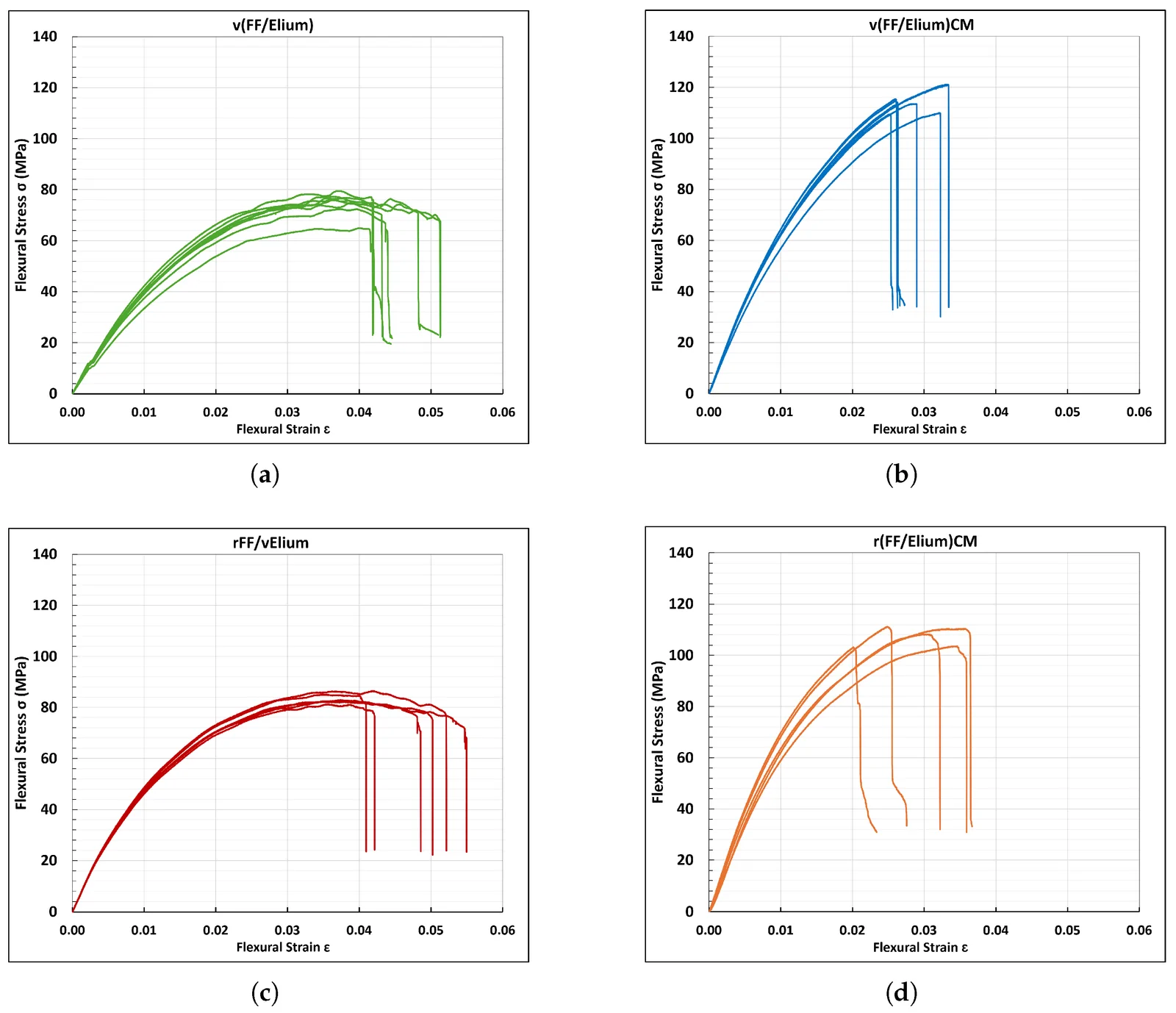
Flexural test results: (**a**) v(FF/Elium^®^), virgin FF with virgin liquid Elium^®^; (**b**) v(FF/Elium^®^)CM, virgin FF with virgin liquid Elium^®^, compression moulded; (**c**) rFF/vElium^®^, reclaimed FF with virgin liquid Elium^®^; and (**d**) r(FF/Elium^®^)CM, reclaimed FF with reclaimed Elium^®^ polymer powder, compression moulded.

The flexural stress/strain curves for the first- and second-generation laminates are shown in Figure 10.

The infused virgin laminate, v(FF/Elium^®^), exhibited the lowest flexural strength, while rFF/vElium^®^ showed similar damage evolution but slightly higher flexural strength. Both the infused laminates (Figure 10a,c) displayed an initial linear bending response followed by progressive nonlinearity, consistent with matrix cracking, fibre/matrix debonding, and fibre pull-out. Failure appeared to initiate predominantly on the tensile face of the specimens under flexural loading. On the other hand, compression moulding led to higher compaction of the laminates, and the flexural stress–strain behaviours (Figure 10b,d) were different from the infused ones.

##### Flexural Strength

**Figure 11:**
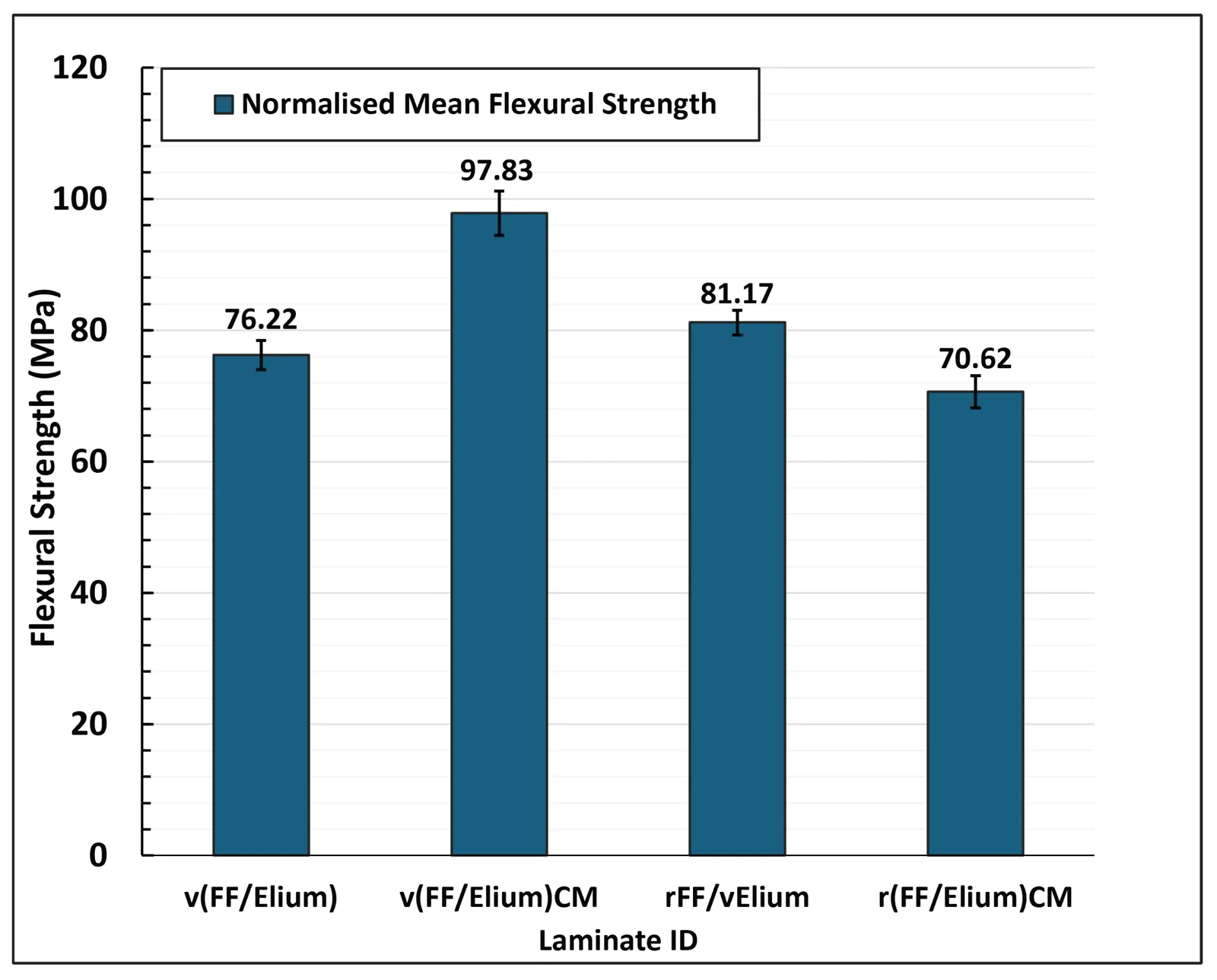
Normalised mean flexural strength of respective samples.

The flexural strength of respective samples is shown in Figure 11. The higher flexural strength of rFF/vElium^®^ compared to v(FF/Elium^®^) (81.17 ± 1.88 MPa vs. 76.22 ± 2.21 MPa) can be attributed to the formation of semi-IPNs at the rFF/matrix interface [21]. Strong interfacial bonds force the matrix and fibre to fail together rather than individually.

The compression-moulded laminates v(FF/Elium^®^)CM and r(FF/Elium^®^)CM present markedly different normalised mean flexural strengths (97.83 ± 3.35 MPa and 70.62 ± 2.46 MPa, respectively). While r(FF/Elium^®^)CM has a nearly 10% higher fibre volume fraction than v(FF/Elium^®^)CM, the lower flexural strength suggests that adequate consolidation was not achieved during compression moulding, likely because the processing temperature and time were insufficient to fully melt and redistribute the reclaimed polymer powder. Despite this, the laminates made with reclaimed materials exhibited comparable or even improved properties relative to those of the virgin laminate, highlighting the potential for successful recycling and reuse without compromising structural performance.

##### Flexural Modulus

**Figure 12:**
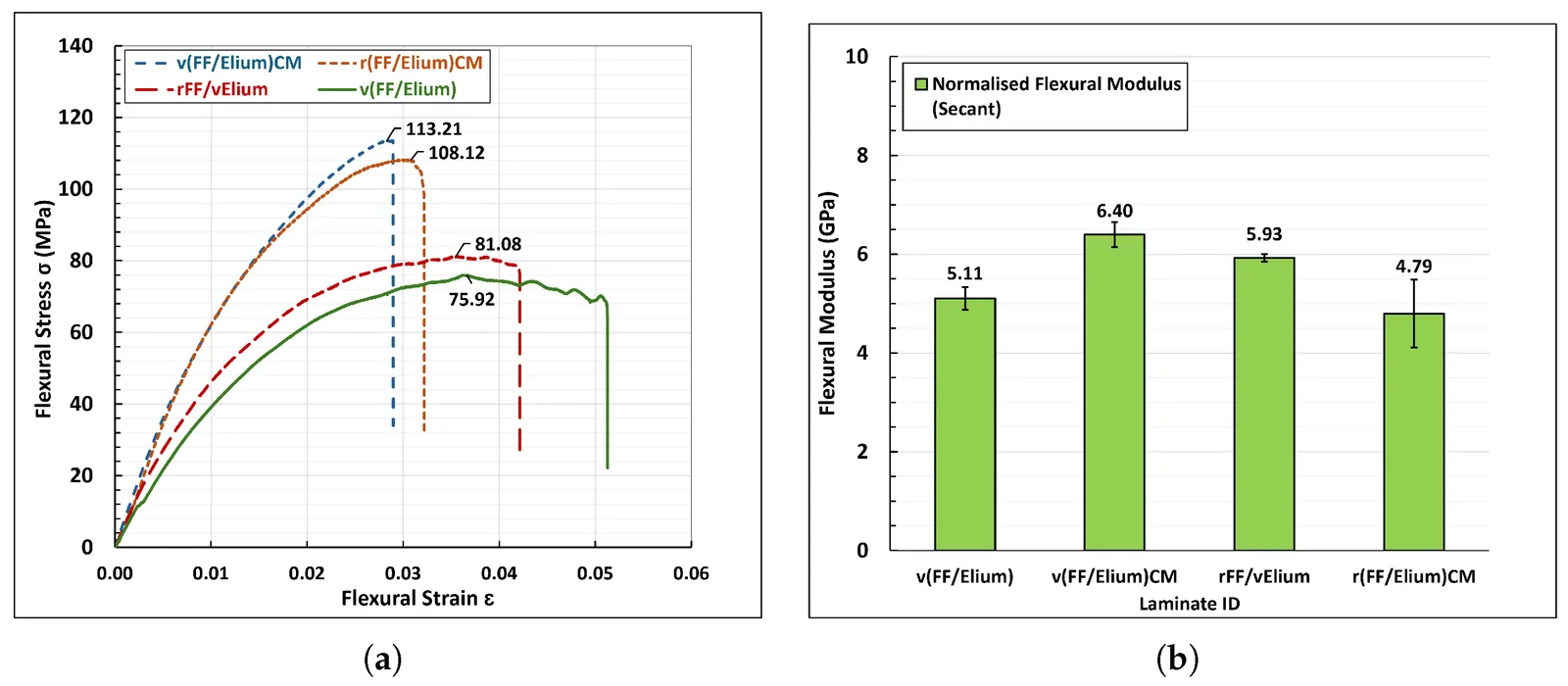
Flexural test results: (**a**) representative best-fit sample curves and (**b**) normalised flexural modulus curves.

Representative best-fit flexural stress–strain and flexural moduli can be seen in Figure 12a,b.

The v(FF/Elium^®^)CM and rFF/vElium^®^ laminates exhibited superior flexural modulus compared to the virgin laminate, with reported moduli 25.3% and 16% higher, respectively. Conversely, the modulus of r(FF/Elium^®^)CM was 6.3% lower, though still comparable to the virgin laminate.

The improvement in rFF/vElium^®^ can again be attributed to the formation of semi-IPNs during in situ polymerisation of rFF, while the superior 25.3% increase in modulus of v(FF/Elium^®^)CM can be attributed to the melting and consolidation of the matrix around the fibres during compression moulding.

Despite the mechanical benefits of postprocessing virgin FF/acrylic with compression moulding, its higher energy demand and associated costs may outweigh the performance gains. Given the substantially lower environmental impact of acetone used in rFF/vElium^®^ compared with high-temperature platen pressing used in v(FF/Elium^®^)CM, the former may present a more attractive processing route.

#### 4.2.2. Short Beam Strength Results

**Figure 13:**
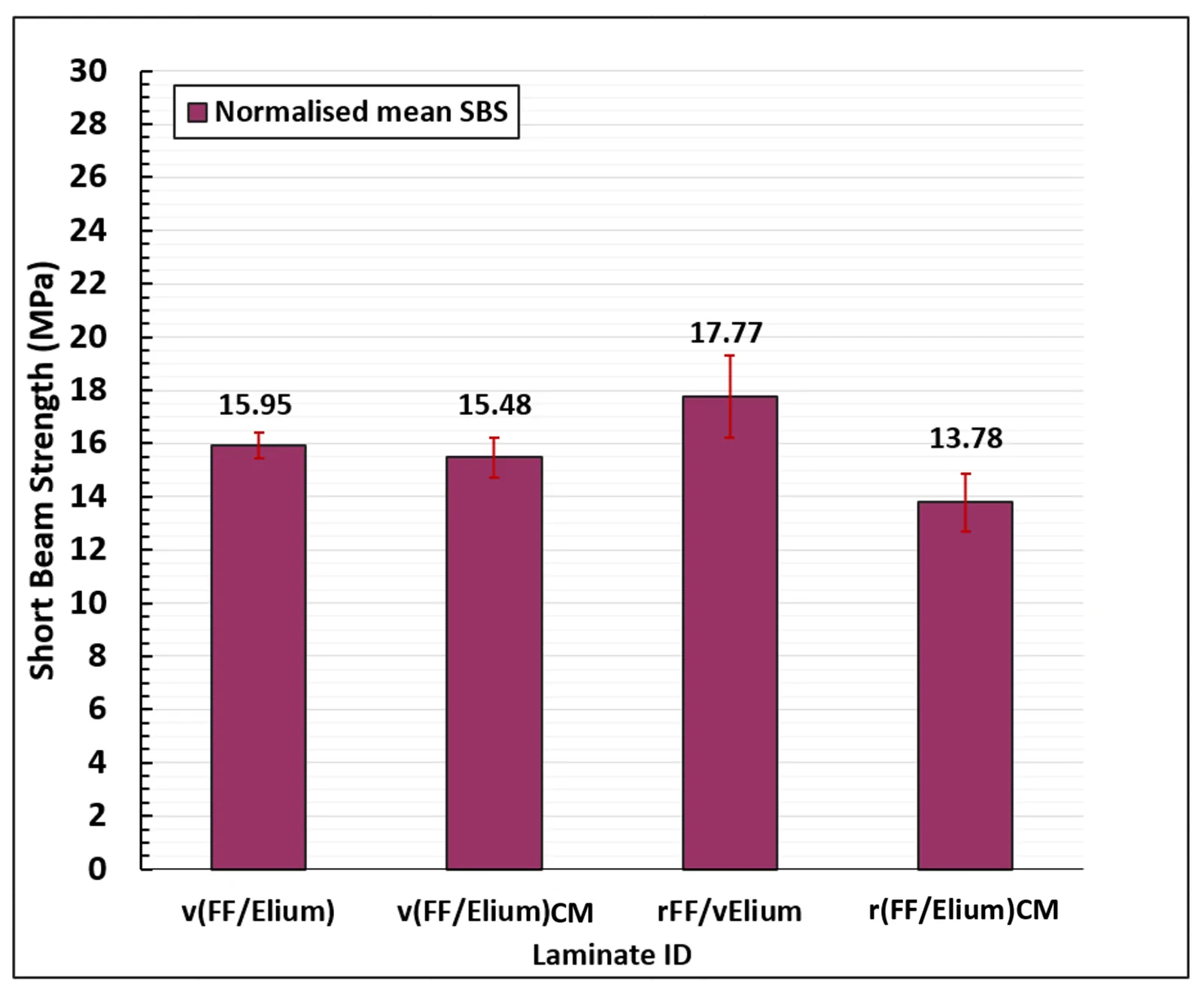
Short beam shear (SBS) strength of respective samples.

The short beam shear strength (SBS) was calculated according to ASTM D2344. The results are displayed in Figure 13.

Comparing the laminates manufactured with VARTM, namely, v(FF/Elium^®^) and rFF/vElium^®^, the latter demonstrated an 11.4% higher normalised short beam strength of 17.77 ± 1.53 MPa, compared to 15.95 ± 0.49 MPa for the former. This is an expected result of semi-IPN formation between the residual polymer found on the reclaimed fabrics and the virgin Elium^®^ resin used in the VARTM process. This suggests that reuse of reclaimed fabrics can be a favourable option in industrial applications when using VARTM without additional fabric treatment (sizing). The residual acrylic polymer left on the reclaimed fibres after the dissolution process acts as an effective sizing agent for the newly infused acrylic resin.

The compression-moulded laminates demonstrated lower yet comparable short beam strength compared to the virgin laminate, with v(FF/Elium^®^)CM and r(FF/Elium^®^)CM displaying short beam strengths of 15.48 ± 0.76 MPa and 13.78 ± 1.07 MPa, respectively. The lower normalised SBS observed in the second-generation compression-moulded laminate compared to that of the virgin laminate can be attributed to insufficient flow of acrylic polymers and inadequate consolidation of the individual layers at 170 °C. Acrylic polymers are often melt-processed around 200 °C, but here the temperature was kept low to avoid thermal degradation of the FF, which limited how much flow could be achieved with the acrylic matrix.

#### 4.2.3. Summary of Mechanical Properties

Table 3 provides the mechanical properties of the tested laminates. All properties are normalised to a $V_{f}$ of 27.37%, that of the reference laminate v(FF/Elium^®^), using Equation (A4) in Appendix D. The as-measured (un-normalised) data are given in Table A4, Appendix E.

#### 4.2.4. Scanning Electron Microscopy (SEM)

**Figure 14:**
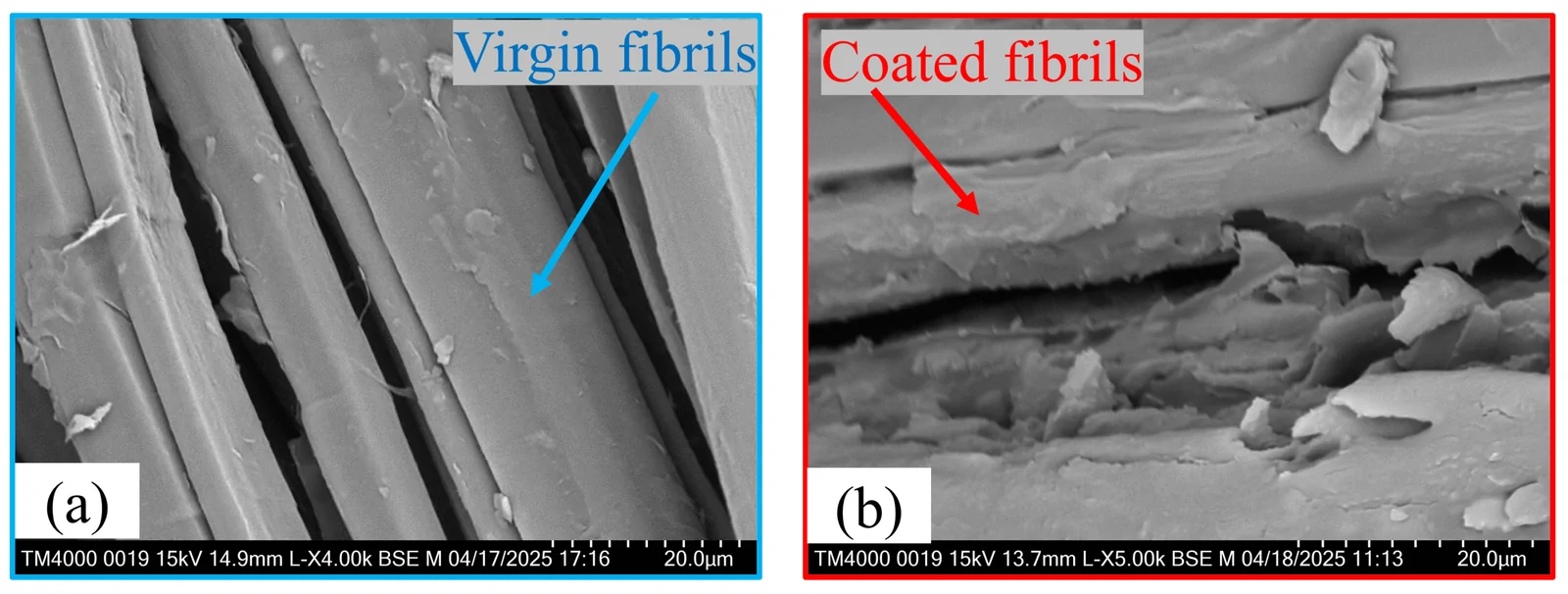
SEM images of fabric surfaces: (**a**) Virgin FF and (**b**) reclaimed FF (rFF).

**Figure 15:**
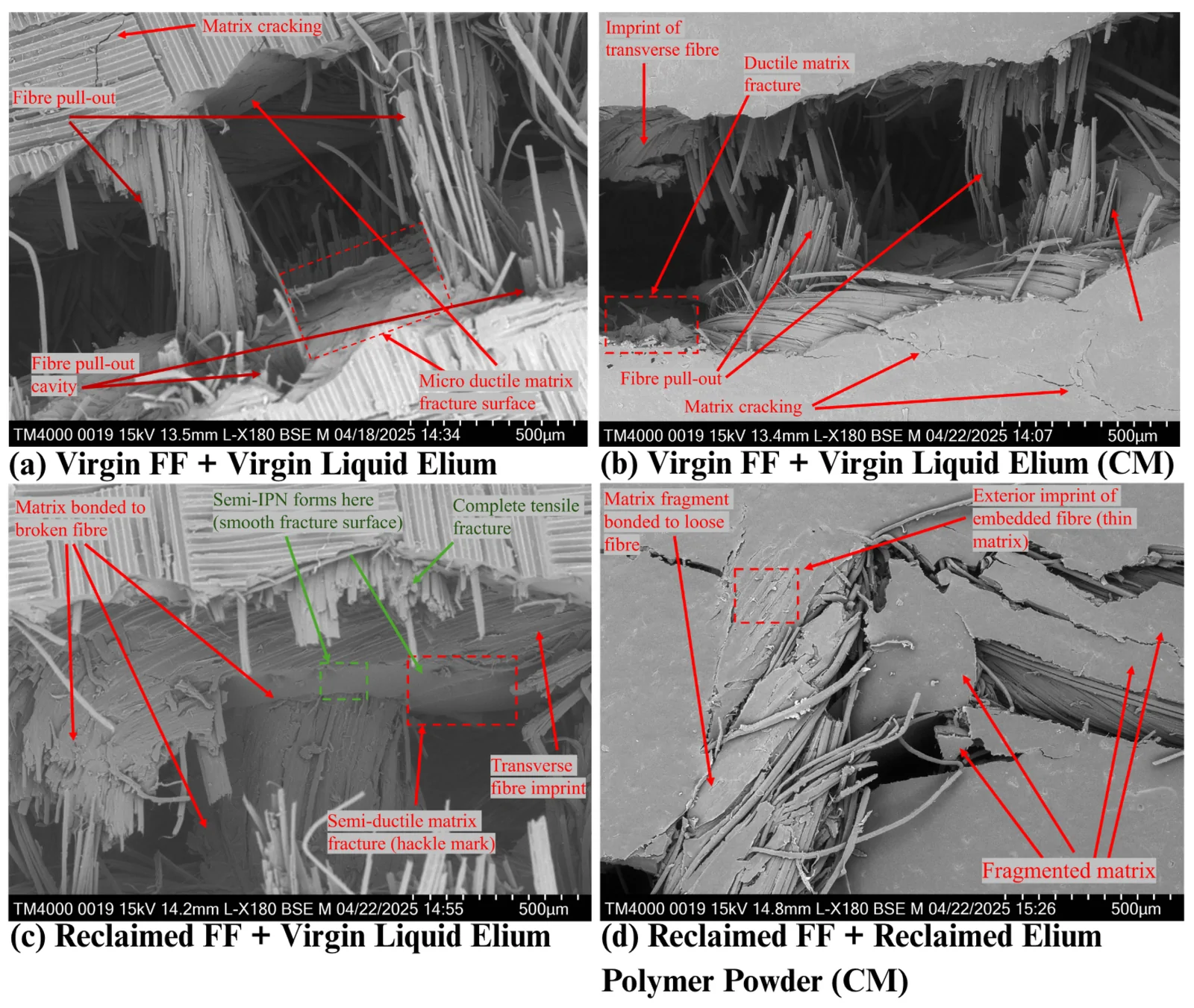
SEM images of flexural sample fracture surfaces: (**a**) v(FF/Elium^®^), (**b**) v(FF/Elium^®^)CM, (**c**) rFF/vElium^®^, (**d**) r(FF/Elium^®^)CM.

Figure 14 compares the fibres in the virgin and reclaimed flax fibre fabric samples. As shown in Figure 14b, the reclaimed fibres are coated with an acrylic matrix residue, a remnant of the dissolution recycling process; this residue promotes the formation of semi-interpenetrating polymer networks (semi-IPNs) [21]. The mass of reclaimed fabrics was 23.3% (1:8 P/S) and 10.1% (1:14 P/S) higher than the virgin fabrics, respectively, due to residual polymer coating after dissolution. The residual polymer changes the surface characteristics of the fabric, leading to an improved mechanical response of the second-generation composites, as shown by the fracture surface SEM images in Figure 15.

Figure 15 compares the fracture surfaces of flexural test specimens for each composite. As seen in Figure 15a, fibre pull-out is significant in the virgin laminate v(FF/Elium^®^). This is expected, as the fibres were not treated with sizing agents [4]. Conversely, it can be seen in Figure 15c that fibre pull-out is reduced for rFF/vElium^®^ compared to the virgin laminate. This suggests that formation of semi-IPNs increased the interfacial bond strength between the reclaimed fabric and the liquid Elium^®^ resin compared to the virgin fabric.

Figure 15b presents evidence of fibre pull-out upon fracture in v(FF/Elium^®^)CM; however, this is less pronounced than in the virgin laminate v(FF/Elium^®^) and more pronounced than in rFF/vElium^®^. This result suggests that compression moulding improved the consolidation quality of the laminate compared to VARTM only, though not to the extent achieved via semi-IPN formation. In Figure 15d, the fracture surface is not clearly visible due to the low fracture strains, and fibre pull-out is not evident.

#### 4.2.5. DMA Results

**Figure 16:**
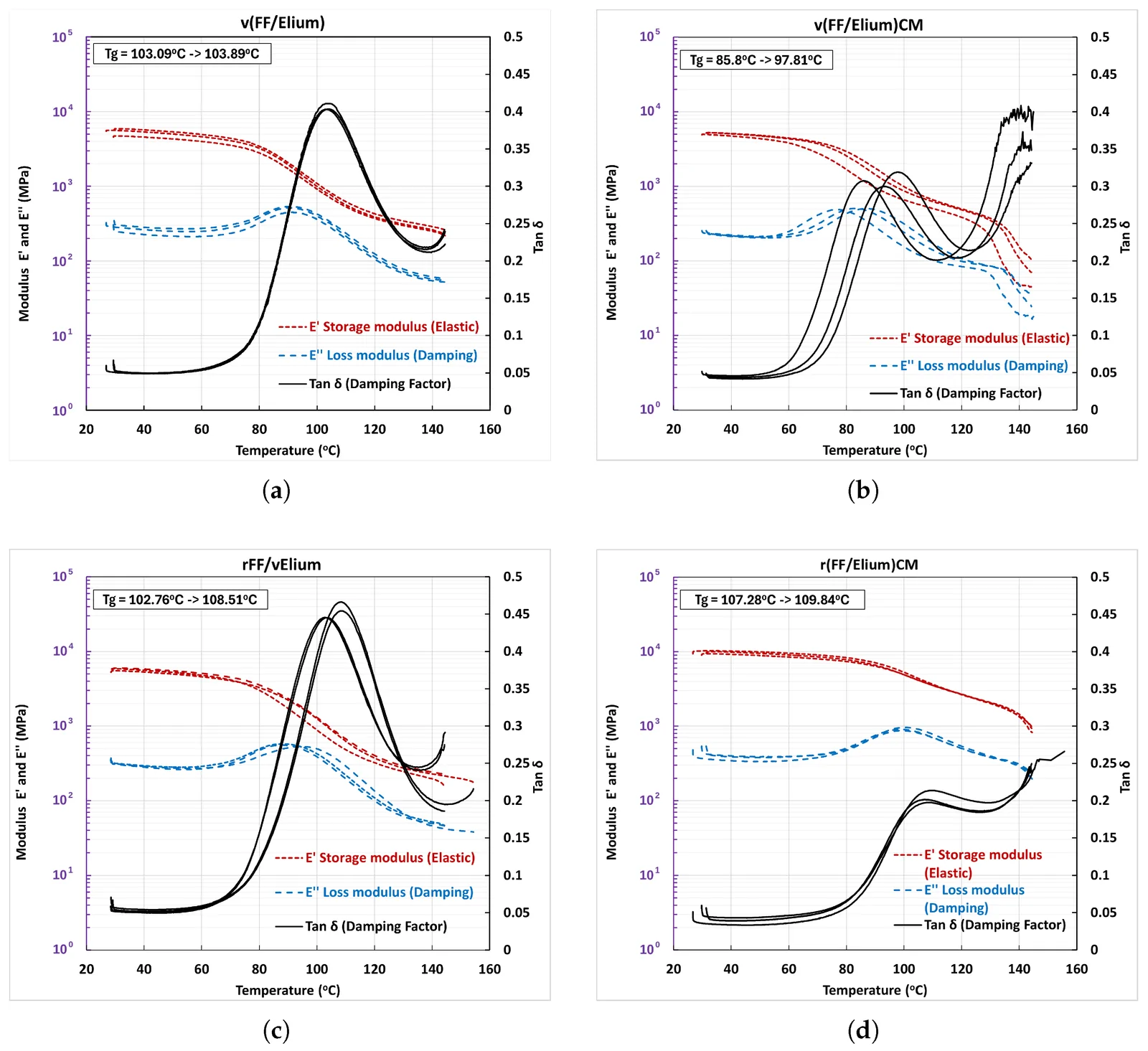
DMA test results: (**a**) v(FF/Elium^®^), virgin FF with virgin liquid Elium^®^; (**b**) v(FF/Elium^®^)CM, virgin FF with virgin liquid Elium^®^, compression moulded; (**c**) rFF/vElium^®^, reclaimed FF with virgin liquid Elium^®^; and (**d**) r(FF/Elium^®^)CM, reclaimed FF with reclaimed Elium^®^ polymer powder, compression moulded.

Figure 16 shows the glass transition temperature ($T_{g}$) of first- and second-generation laminates, measured at the maximum tan δ. The range is relatively consistent for v(FF/Elium^®^), rFF/vElium^®^, and r(FF/Elium^®^)CM, all falling between 103 °C and 110 °C; however, v(FF/Elium^®^)CM has a significantly lower range, with a $T_{g}$ between 86 °C and 98 °C (Figure 16b). A comparison between the storage modulus and the experimental flexural modulus can be seen in Table 4.

While both r(FF/Elium^®^)CM and v(FF/Elium^®^)CM experienced two consecutive heat cycles (compression moulding, then DMA), the compression moulding process was carried out at higher pressure (35 bar vs. 20 bar) and higher temperature (170 °C vs. 150 °C) when manufacturing r(FF/Elium^®^)CM. Higher temperature stiffens the fibres in r(FF/Elium^®^)CM, as suggested by previous studies on heat treatment of flax fibres [16], while higher pressure increases $V_{f}$ (41.59% vs. 31.86%).

The higher $V_{f}$ in r(FF/Elium^®^)CM leads to elastic behaviour dominating for a wider temperature range, as seen in Figure 16d, where the storage modulus begins to gradually decrease from ≈10.2 GPa between 90 °C and 100 °C, versus the sudden decrease from ≈5.3 GPa between 80 °C and 90 °C in v(FF/Elium^®^)CM seen in Figure 16b. The higher $V_{f}$ results in increased stiffness, and so the storage modulus, of the r(FF/Elium^®^)CM laminate; in addition, it reduces the tan δ peak and shifts it to a higher temperature. However, it is also important to note that the peak tan δ is higher for v(FF/Elium^®^)CM than for r(FF/Elium^®^)CM (≈0.3 vs. ≈0.2). This suggests that v(FF/Elium^®^)CM has better damping and vibration absorption properties, which can be useful in non-structural applications.

The lower $T_{g}$ and storage modulus of v(FF/Elium^®^)CM might be attributed to the compression moulding process causing the molecules to rearrange and freeze in a different amorphous state. In the case of r(FF/Elium^®^)CM, where the temperature and pressure were even higher, a molecular arrangement also occurred; however, there was an additional interaction at the fibre/matrix interface due to the presence of the residual matrix on the fibre, which led to the high $T_{g}$. For an amorphous polymer, different processing temperature and pressure histories—particularly cooling under pressure—can result in different molecular packing, free volume, and chain mobility. In the presence of fibres, fibre/matrix interfacial interactions may further constrain the chains and alter the local molecular arrangement. Consequently, these differences in the amorphous structure and segmental mobility can lead to variations in the measured $T_{g}$. This might be the reason behind the different $T_{g}$ values observed in v(FF/Elium^®^)CM and r(FF/Elium^®^)CM.

A key observation is that the tail of the tan δ curve is steeper in v(FF/Elium^®^)CM than r(FF/Elium^®^)CM. This is indicative of the fact that it becomes more flexible on crossing its $T_{g}$, while r(FF/Elium^®^)CM retains its rigidity to a higher extent even after crossing $T_{g}$. This is supported by the observation that the $T_{g}$ of r(FF/Elium^®^)CM is 12 °C higher than v(FF/Elium^®^)CM.

In terms of the infused laminates v(FF/Elium^®^) and rFF/vElium^®^, it is clear that the recycling process had a nearly negligible impact on the operating temperature of the composites, which are safe to use up to 60 °C to 65 °C. Figure 16c suggests that the $T_{g}$ of rFF/vElium^®^ could potentially reach up to 108.5 °C, compared to 103.5 °C of the virgin laminate. This slight increase in $T_{g}$ can be attributed to the formation of semi-IPNs at the fibre–matrix interface. These semi-IPNs limit chain mobility, requiring more thermal energy (higher temperatures) for the damping forces to dominate.

### 4.3. Molecular Simulation Results

Simulations of the isolated phases (cellulose, liquid monomer, and PMMA) yielded good agreement with experimental density values. For liquid methyl methacrylate, the obtained density was 0.956 ± 0.005 g/cm^3^ (experimental value: 0.944 g/cm^3^ [39]), while for PMMA it was 1.193 ± 0.007 g/cm^3^ (experimental value: 1.18 g/cm^3^ [40]). For amorphous cellulose, a density of 1.340 ± 0.007 g/cm^3^ was obtained. This value was estimated to be consistent with the properties of the experimental flax fibre samples used in this work, as follows: the flax fibre has a density of 1.45 g/cm^3^ with 9% water content; excluding the water component results in a semicrystalline polymer density of 1.52 g/cm^3^. This value, together with the density of the amorphous phase obtained from the simulations and a density of 1.6 g/cm^3^ [41] for the cellulose Iβ crystalline phase, yields an approximate crystalline fraction of 0.72, which is within the expected range.

**Figure 17:**
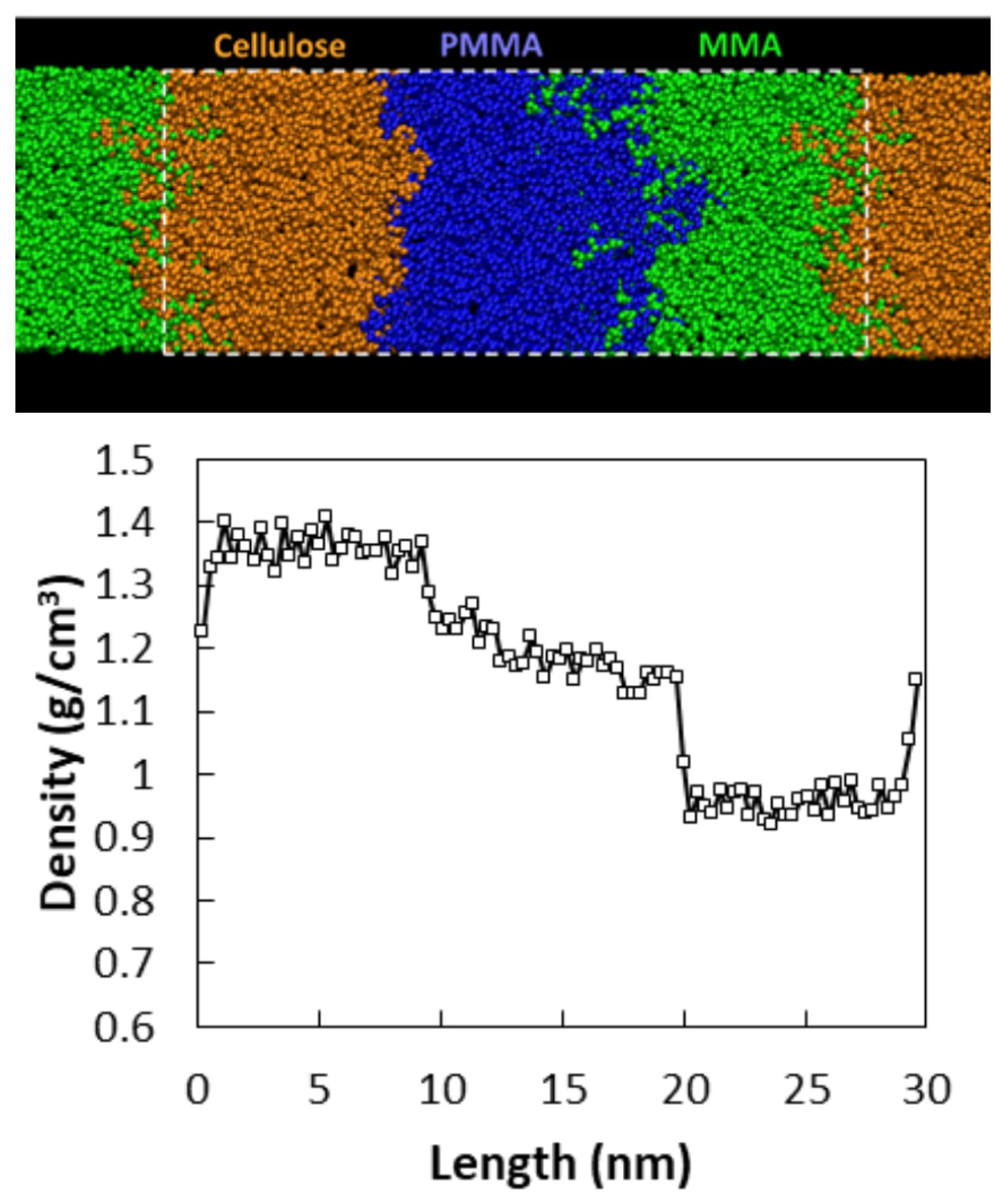
Final configuration of the molecular model of cellulose/PMMA/MMA interfaces and density profile.

Figure 17 shows the final configuration of the simulated ternary system and its density profile. It can be observed that the PMMA phase (blue) reaches a higher density near the cellulose phase (orange). This effect extends over a depth of approximately 2.5 nm, beyond which the density approaches the bulk value obtained from simulations of the isolated phases. Such near-surface densification is characteristic of limited interfacial interactions, where polymer chains become oriented and confined in the vicinity of the interface [42].

This behaviour is supported by the calculated nonbonded interaction energy per unit area, $E_{ij}$, between atoms belonging to different phases (e.g., cellulose with PMMA or MMA). These provide a qualitative indicator of relative interfacial affinity, with lower (more negative) values indicating stronger interaction.

The calculated interaction energies were $E_{\mathrm{PMMA}/\mathrm{MMA}}=-0.39\;\mathrm{kcal}/\mathrm{mol}/{Å}^{2}$, $E_{\mathrm{cellulose}/\mathrm{MMA}}=-0.25\;\mathrm{kcal}/\mathrm{mol}/{Å}^{2}$, and $E_{\mathrm{PMMA}/\mathrm{cellulose}}=-0.23\;\mathrm{kcal}/\mathrm{mol}/{Å}^{2}$. The value obtained for the cellulose/MMA interface is comparable to that of PMMA/cellulose.

Interestingly, it was observed that liquid MMA penetrates more deeply into the PMMA phase than into the cellulose phase, which may facilitate interpenetration between the residual acrylic coating and freshly infused MMA resin during subsequent polymerisation. The simulation results are in line with the observed mechanical performance of the second-generation composites, particularly rFF/vElium^®^, where a noticeable increase in short beam shear strength is indicative of the stronger interfacial affinity ($E_{\mathrm{PMMA}/\mathrm{MMA}}=-0.39\;\mathrm{kcal}/\mathrm{mol}/{Å}^{2}$) between coated fibres and fresh resin (the PMMA and MMA, respectively) compared to the virgin composite ($E_{\mathrm{cellulose}/\mathrm{MMA}}=-0.25\;\mathrm{kcal}/\mathrm{mol}/{Å}^{2}$), as seen in Figure 13. Furthermore, there is evidence of the formation of semi-IPNs in the SEM images (Figure 15c). Simulations were performed at static equilibrium, not under loading; therefore, they do not directly provide information about the mechanical properties of the system. In future work, it would be interesting to further investigate these effects by performing simulations under loading conditions, as well as to probe the stress profile and mechanical response of the different interface types.

## 5. Comparative Analysis of FF/Thermoplastic Mechanical Properties

The second generation Elium^®^ 191 flax fibre (FF) composites demonstrate promising performance when benchmarked against similar first generation virgin flax fibre (FF) reinforced polymer composites, as seen in Table 5.

In the flexural strength analysis, v(FF/Elium^®^)CM demonstrated the highest strength of the four laminates characterised in this study at 113.88 MPa, followed by r(FF/Elium^®^)CM at 107.30 MPa. These values are comparable to the flax fibre composite systems given in Table 5, most notably to thermoset vinyl ester (108.7 MPa), and outperform the bio-based lactic acid thermoset (PMLA) composites (50.46 MPa to 86.2 MPa). This suggests that reprocessing through compression moulding of Elium^®^ 191 can match or exceed the performance of traditional thermoset resins in flexural strength. While the flexural strengths of v(FF/Elium^®^)CM and r(FF/Elium^®^)CM are surpassed by hand layup epoxy (131.3 MPa) and RTM woven FF/vinyl ester (130 MPa), the thermoplastic laminates achieve ≈82–88% of their flexural strength. A similar trend is seen with the flexural moduli of the composites.

The flexural strengths of v(FF/Elium^®^) and rFF/vElium^®^ (76.22 and 83.44 MPa) exceed woven FF/PLA 3025D (47.18 MPa) and fall within the range reported for woven FF/PMLA systems (43.34–86.2 MPa).

For the short beam shear strength (SBS), the r(FF/Elium^®^)CM laminate exhibited the highest SBS among all examined composites at 20.94 MPa, exceeding that of unidirectional acrylic (UD) systems as well as alkali-treated flax (A Flax) composites. This suggests that reprocessing can enhance interfacial bonding, potentially eliminating the need for additional fibre surface treatments in flax–acrylic systems. In rFF/vElium^®^ and r(FF/Elium^®^)CM, the residual acrylic polymer remaining on the fabrics after dissolution likely acts as a sizing agent, promoting semi-IPN formation at the fibre–matrix interface.

The first- and second-generation laminates exhibited very comparable performance to that of equivalent virgin flax fibre-based thermoplastic laminates reported in previous papers. The use of reclaimed materials was not found to have any significant detrimental effect on the performance of the second-generation laminates.

## 6. Conclusions

This paper has investigated the recycling of flax fibre/acrylic composites via dissolution along with the properties of both first- and second-generation laminates. Two first-generation (virgin) flax fibre/Elium^®^ laminates were benchmarked against two second-generation laminates produced using different recycling and reprocessing routes, including different levels of reclaimed materials. Dissolution enabled separation of the acrylic polymer matrix from the woven flax fibre reinforcement using acetone. A key novelty of the process in this paper is precipitation of the dissolved acrylic polymer using water alone as the non-solvent, which achieved 80% polymer recovery. Furthermore, the continuous form of the flax reinforcement was preserved; this addresses a major limitation of many conventional recycling methods, which often require chopping of larger parts.

The second-generation laminates showed comparable or improved flexural and short beam strength properties relative to the virgin laminate when comparing un-normalised measured properties. After normalisation with respect to $V_{f,\mathrm{ref}}$, the infused rFF/vElium^®^ laminate retained its improved flexural and short beam strength, whereas r(FF/Elium^®^)CM, the fully-reclaimed compression moulded laminate, showed lower normalised values. These results indicate that further optimisation of polymer powder redistribution and consolidation are still required. Dynamic mechanical analysis showed that recycling had a negligible effect on the glass transition temperature of the second-generation composites, while the first-generation v(FF/Elium^®^)CM laminate produced by compression moulding exhibited a reduction of approximately 10%. The retention of $T_{g}$ in the second-generation laminates is consistent with semi-IPN formation at the fibre/matrix interface, which contributes to the improved properties, whereas the reduction of $T_{g}$ for v(FF/Elium^®^)CM can be attributed to its processing history; the absence of a comparable reduction in r(FF/Elium^®^)CM suggests that semi-IPN formation offsets this effect in the second-generation laminate.

The above experimental findings and the molecular simulations were consistent, suggesting that the formation of semi-IPNs contributed significantly to the improved mechanical performance of the recycled laminates. These results demonstrate that the proposed recycling routes can be used to recover and re-manufacture flax fibre/acrylic composites without compromising their structural performance, and potentially even improving it. In future studies, it would be interesting to investigate how this works with synthetic reinforcements such as glass or carbon fibre.

Overall, the recycling strategy developed in this work addresses several important limitations of conventional composite recycling routes. Although natural fibres are inherently biodegradable, this advantage is largely lost when they are combined with synthetic polymer matrices, making effective recycling routes essential for improving material circularity. In contrast to thermal or mechanical recycling methods such as pyrolysis and incineration, which are inherently energy intensive and often require parts to be reduced in size prior to processing, the dissolution route developed here with water used as a non-solvent is performed at room temperature and offers greater potential for industrial scalability with natural fibre composites. Furthermore, by preserving the continuity of the flax fibre reinforcement and recovering the acrylic polymer through water-induced precipitation, the process supports reuse in larger second-generation composite parts. This makes the approach in this paper particularly attractive for applications such as the automotive or marine industries, where large component dimensions, low-energy processing, and material circularity are of importance due to high production volumes. However, the dissolution process, which was conducted over 68 h in this study, may not be ideal for an industrial setup; both the dissolution time and the P/S ratio require further optimisation. To understand the effect of the P/S ratio on the recovered acrylic polymer, future studies should characterise polymers recovered at different P/S ratios in order to determine whether the glass transition temperature ($T_{g}$) shifts as a result of changes in chain relaxation and entanglement. Further research is also needed to establish the most effective and efficient recovery process.

## Figures and Tables

**Table 1:** Summary of laminate identification codes and processing history. VARTM = vacuum-assisted resin transfer moulding (liquid resin infusion); CM = compression moulding; v = virgin; r = reclaimed. Note that v(FF/Elium^®^)CM is obtained by liquid resin infusion followed by compression moulding of v(FF/Elium^®^).

ID	Generation	Process	Description
v(FF/Elium^®^)	First	VARTM	Virgin FF/Elium^®^ laminate produced by liquid resin infusion
v(FF/Elium^®^)CM	First	VARTM->CM	Compression-moulded laminate made from virgin FF/Elium^®^
rFF/vElium^®^	Second	VARTM	Reclaimed FF infused with virgin liquid Elium^®^ resin
r(FF/Elium^®^)CM	Second	CM	Compression-moulded laminate using reclaimed flax fibres and reclaimed Elium^®^ polymer powder

**Table 2:** Laminate fibre volume fractions ($V_{f}$).

Laminate ID	$V_{f}$	Thickness (mm)
v(FF/Elium^®^)	27.37%	3.30
v(FF/Elium^®^)CM	31.86%	2.95
rFF/vElium^®^	28.14%	3.35
r(FF/Elium^®^)CM	41.59%	2.25

**Table 3:** Normalised mechanical properties of the tested laminates, including mean flexural strength, mean SBS, and modulus. Outlier specimens were excluded from the calculated properties.

Laminate ID	Flexural Strength	Short Beam Strength	Flexural Modulus
	[MPa]	[MPa]	[GPa]
v(FF/Elium^®^)	76.22 ± 2.21	15.95 ± 0.49	5.11 ± 0.23
v(FF/Elium^®^)CM	97.83 ± 3.35	15.48 ± 0.76	6.40 ± 0.25
rFF/vElium^®^	81.17 ± 1.88	17.77 ± 1.53	5.93 ± 0.08
r(FF/Elium^®^)CM	70.62 ± 2.46	13.78 ± 1.07	4.79 ± 0.69

**Table 4:** Summary of dynamic mechanical analysis. The storage modulus and flexural modulus are normalised to a $V_{f}$ of 27.37% using Equation (A4). $E^{\prime }$ is reported at 32 °C, the lowest temperature common to every temperature ramp. Normalised flexural modulus values are reproduced from Table 3. As-measured values are given in Table A5.

Laminate ID	Storage Modulus $E^{\prime }$ at 32 °C	Flexural Modulus	$T_{g}$ (Peak Tan δ)	Peak Tan δ
	[GPa]	[GPa]	[°C]	[–]
v(FF/Elium^®^)	5.38 ± 0.61	5.11 ± 0.23	103.5 ± 0.4	0.406 ± 0.004
v(FF/Elium^®^)CM	4.42 ± 0.15	6.40 ± 0.25	092.3 ± 6.1	0.309 ± 0.010
rFF/vElium^®^	5.68 ± 0.25	5.93 ± 0.08	105.8 ± 3.1	0.453 ± 0.010
r(FF/Elium^®^)CM	6.48 ± 0.29	4.79 ± 0.69	108.6 ± 1.3	0.204 ± 0.008

**Table 5:** Flexural and interlaminar shear properties of flax fibre composites with different matrix systems and processing routes (as-measured un-normalised values). Subscripts: a—v(FF/Elium^®^); b—v(FF/Elium^®^)CM; c—rFF/vElium^®^; d—r(FF/Elium^®^)CM.

Fibre	Matrix	Matrix Type	v/wt	Flex. E	Flex. Strength	SBS	Proc. Method	Src.
			[%]	[GPa]	[MPa]	[MPa]		
Flax Woven	Vinyl Ester	TS	35 v	8.7	130	≈14	RTM	[43]
Flax Woven	Vinyl Ester	TS	34 v	6.7	108.7	[-]	VARTM	[44]
Flax Woven	PMLA	TS (low vis.)	40 wt	9.9	86.2	[-]	CM	[45]
Flax Woven	PMLA	TS (low vis.)	50 wt	6.38	43.34	[-]	CM	[45]
Flax Woven	PMLA	TS (low vis.)	60 wt	6.72	50.46	[-]	CM	[45]
Flax Woven	Epoxy	TS	[-]	6.21	131.3	[-]	Hand Layup	[46]
Flax UD	Elium^®^ 150 (Acrylic)	TP resin	40–45 v	[-]	[-]	18.5	VARTM	[4]
A Flax UD	Elium^®^ 150 (Acrylic)	TP resin	40–45 v	[-]	[-]	20.4	VARTM	[4]
PDA Flax UD	Elium^®^ 150 (Acrylic)	TP resin	40–45 v	[-]	[-]	19.0	VARTM	[4]
Flax Woven	PLA 3025D	TP film	35 v	7.82	47.18	[-]	CM	[47]
Flax Woven	Elium^®^ 191 (Acrylic)	TP resin	27 v	5.11	76.22	15.95	VARTM	[a] this study
Flax Woven	Elium^®^ 191 (Acrylic)	TP resin	32 v	7.45	113.88	18.02	VARTM → CM	[b] this study
Flax Woven	Elium^®^ 191 (Acrylic)	TP resin	28 v	6.09	83.44	18.27	VARTM (2nd gen)	[c] this study
Flax Woven	Elium^®^ 191 (Acrylic)	TP powder	42 v	7.28	107.30	20.94	CM (2nd gen)	[d] this study

Abbreviations: TS—thermoset; TP—thermoplastic; PMLA—pentaerythritol-modified methacrylated lactic acid resin; PLA—polylactic acid; UD—unidirectional; A Flax—alkali-treated flax; PDA Flax—poly(dopamine)-coated flax; RTM—resin transfer moulding; VARTM—vacuum assisted resin transfer moulding; CM—compression moulding; SBS—short beam strength; low vis.—low viscosity. Fibre content is given as a volume fraction (v) or a weight fraction (wt), as reported in the cited source. Entries marked [-] were not reported.

## Data Availability

The original contributions presented in this study are included in the article. Further inquiries can be directed to the corresponding author.

## Abbreviations

The following abbreviations are used in this manuscript:

CF	Carbon fibre
CM	Compression moulding
DMA	Dynamic mechanical analysis
EU	European Union
FF	Flax fibre
HPC	High-performance computing
LAMMPS	Large-scale Atomic/Molecular Massively Parallel Simulator
MAPS	Materials and Processes Simulations platform
MEKP	Methyl ethyl ketone peroxide
MMA	Methyl methacrylate
NPT	Isothermal–isobaric ensemble
NVT	Canonical ensemble
PDA	Poly(dopamine)
PET	Poly(ethylene terephthalate)
PLA	Polylactic acid
PMLA	Pentaerythritol-modified methacrylated lactic acid
PMMA	Poly(methyl methacrylate)
PP	Polypropylene
PPPM	Particle–particle particle–mesh
P/S	Polymer-to-solvent ratio
RTM	Resin transfer moulding
SBS	Short beam shear (strength)
SEM	Scanning electron microscopy
semi-IPN	Semi-interpenetrating polymer network
TP	Thermoplastic
TS	Thermoset
UD	Unidirectional
VARTM	Vacuum-assisted resin transfer moulding
VMD	Visual Molecular Dynamics
$V_{f}$	Fibre volume fraction

## Appendix A. Reinforcing Fabric Properties

**Table A1:** Mechanical properties of flax fabric in the weft and warp directions.

Property	Directional Data		Unit
	Weft	Warp	
Tensile strength	75 ± 4	78 ± 2	MPa
Young’s modulus	5 ± 1	6 ± 1	GPa
Elongation at break	2.4 ± 0.2	3.2 ± 0.2	%
Thickness of 1 impregnated ply	0.83 ± 0.02		mm

## Appendix B. Dissolution Mass Balance

The balance reported here is for the 1:14 polymer/solvent route, on which precipitation was performed; for the 1:8 route, only the reclaimed fabric mass gain (23.3%) was recorded. After dissolution, a ternary solution of water, acetone, and acrylic polymer remained. The corresponding mass fractions are presented in Table A2. Approximately 9 g of acrylic polymer remained as residue on the reclaimed fabrics, leaving 131.2 g of polymer dissolved in the solution. Of this dissolved fraction, 111 g of polymer was successfully precipitated upon the addition of water as a non-solvent. Further optimisation is required to enable efficient separation and recovery of acetone from the aqueous phase, allowing solvent reuse through fractional distillation [24,25,26,27].

**Table A2:** Mass and yield metrics from the dissolution and precipitation process for a 1:14 polymer/solvent ratio. Tap water was used as the non-solvent.

Parameter	1:14 p/s Ratio
Pre-dissolution laminate polymer mass	140.20 g
Pre-dissolution laminate fabric mass	89.20 g
Mass of acetone	1900.88 g (2406.18 mL)
**Polymer/solvent ratio**	**1:13.56**
Mass of non-solvent (water)	5500 g
Dissolution time	68 h
**Non-solvent/solvent ratio**	**2.89**
Mass of reclaimed fabrics	98.20 g
Mass of polymer in solution	131.20 g
Mass of reclaimed polymer	111.00 g
Mass of residue polymer on fabrics	9.00 g
Total reclaimed polymer	120.00 g
Polymer not recovered (retained in filtrate)	20.20 g
Precipitate yield (solution only)	84.60%
Precipitate yield (total polymer mass)	79.17%
**Residue polymer (on fabrics)**	**6.42%**
**Total polymer yield**	**85.59%**
Polymer loss (unprecipitated)	14.41%
Fibre mass recovered	89.20 g (100%)
**Overall material utilisation**	**91.19%**

## Appendix C. Fibre Volume Fraction Determination

The fibre volume fraction of each laminate was determined from the mass of dry fabric and the mass of the consolidated laminate. The fibre mass fraction $W_{f}$ is given by

(A1) $$W_{f}=\frac{M_{f}}{M_{c}},$$

where $M_{f}$ is the total mass of dry fabric in the layup and $M_{c}$ the mass of the consolidated laminate. Converting from a mass to a volume basis using the constituent densities gives

(A2) $$V_{f}=\frac{\frac{W_{f}}{\rho_{f}}}{\frac{W_{f}}{\rho_{f}}+\frac{1-W_{f}}{\rho_{m}}},$$

and the matrix volume fraction follows as

(A3) $$V_{m}=1-V_{f},$$

with $\rho_{f}=1.45$ g/cm^3^ for the flax fabric (as supplied, including its equilibrium moisture content) and $\rho_{m}=1.01$ g/cm^3^ for the cured acrylic matrix.

Equations (A1)–(A3) treat the laminate as a two-phase solid and so assume zero void content; as no void correction was applied, the reported values should be read as nominal fibre volume fractions. The calculation was performed on three samples of each laminate and the results were averaged. The measured mass fractions and the corresponding volume fractions are given in Table A3.

**Table A3:** Measured fibre mass fractions, corresponding fibre volume fractions obtained from Equation (A2), and the scaling factor applied to each laminate when normalising the mechanical properties (Appendix D).

Laminate ID	$W_{f}$ [%]	$V_{f}$ [%]	$V_{f,\mathrm{ref}}/V_{f}$ [–]
v(FF/Elium^®^)	35.11	27.37	1.000
v(FF/Elium^®^)CM	40.17	31.86	0.859
rFF/vElium^®^	35.99	28.14	0.973
r(FF/Elium^®^)CM	50.55	41.59	0.658

## Appendix D. Normalisation of Mechanical Properties

Mechanical properties were normalised to the fibre volume fraction of the virgin laminate, v(FF/Elium^®^). Each mean property *X* was scaled linearly with fibre volume fraction according to

(A4) $$X_{\mathrm{norm}}=X_{\mathrm{meas}}\times \frac{V_{f,\mathrm{ref}}}{V_{f}},$$

where $X_{\mathrm{meas}}$ is the as-measured mean value for a given laminate, $V_{f}$ its fibre volume fraction determined using Equation (A2), and $V_{f,\mathrm{ref}}=27.37\%$ is the reference value of the virgin laminate. The resulting scaling factors are listed in the final column of Table A3. The glass transition temperature and the peak tan δ are ratio quantities, and so are unaffected by the normalisation.

## Appendix E. Un-Normalised Mechanical Properties

**Table A4:** Un-normalised flexural and short beam properties of the tested laminates.

Laminate ID	$V_{f}$	Flexural Strength	Short Beam Strength	Flexural Modulus
	[%]	[MPa]	[MPa]	[GPa]
v(FF/Elium^®^)	27.37	76.22 ± 2.21	15.95 ± 0.49	5.11 ± 0.23
v(FF/Elium^®^)CM	31.86	113.88 ± 3.90	18.02 ± 0.88	7.45 ± 0.29
rFF/vElium^®^	28.14	83.44 ± 1.93	18.27 ± 1.58	6.09 ± 0.08
r(FF/Elium^®^)CM	41.59	107.30 ± 3.48	20.94 ± 1.63	7.28 ± 1.05

**Table A5:** Un-normalised dynamic mechanical analysis results. The storage modulus and flexural modulus are given at each laminate’s own fibre volume fraction; $T_{g}$ and peak tan δ are ratio quantities and are identical to those reported in Table 4. The corresponding normalised moduli are given in Table 4.

Laminate ID	Storage Modulus $E^{\prime }$ at 32 °C	Flexural Modulus	$T_{g}$ (Peak Tan δ)	Peak Tan δ
	[GPa]	[GPa]	[°C]	[–]
v(FF/Elium^®^)	5.38 ± 0.61	5.11 ± 0.23	103.5 ± 0.4	0.406 ± 0.004
v(FF/Elium^®^)CM	5.14 ± 0.17	7.45 ± 0.29	92.3 ± 6.1	0.309 ± 0.010
rFF/vElium^®^	5.84 ± 0.26	6.09 ± 0.08	105.8 ± 3.1	0.453 ± 0.010
r(FF/Elium^®^)CM	9.85 ± 0.44	7.28 ± 1.05	108.6 ± 1.3	0.204 ± 0.008
